# Supplementary material for: Association between socioeconomic status and self-reported, tested and diagnosed COVID-19 status during the first wave in the Northern Netherlands: a general population-based cohort from 49 474 adults
Source: BMJ Open. 2021 Mar 22;11(3):e048020. doi: 10.1136/bmjopen-2020-048020 (PMC7985974; doi:10.1136/bmjopen-2020-048020)
Supplement: Supplementary data [file bmjopen-2020-048020supp001.pdf]

Supplementary Figure 1. Study flow chart

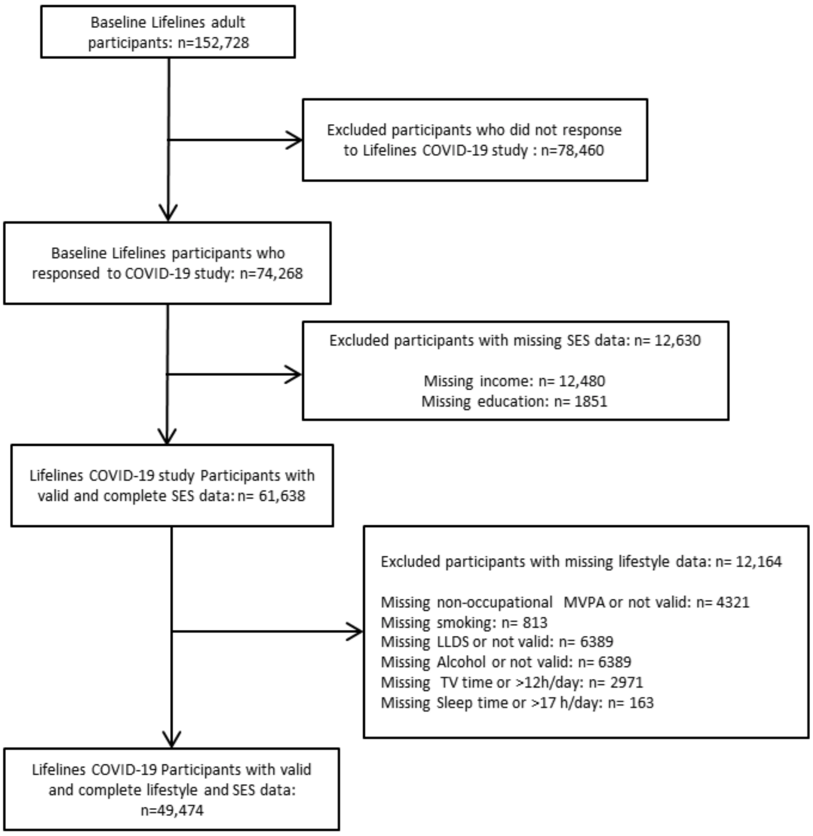

Supplementary Table 1. Association of education or income with COVID-19 status with further adjustment for number of questionnaires filled.

Education	COVID-19 Status					
	Self-reported		Tested		Diagnosed	
	OR [95%CI]	p	OR [95%CI]	p	RR [95%CI]	p
Model 1						
low	0.78 [0.71-0.86]	**<0.001**	0.58 [0.52-0.66]	**<0.001**	1.10 [0.60-2.01]	0.8
middle	0.90 [0.84-0.97]	**0.004**	0.72 [0.66-0.79]	**<0.001**	1.77 [1.21-2.60]	**0.003**
high			Ref			
Model 2						
low	0.79 [0.72-0.87]	**<0.001**	0.61 [0.54-0.68]	**<0.001**	1.08 [0.60-1.95]	0.8
middle	0.91 [0.84-0.97]	**0.006**	0.73 [0.67-0.80]	**<0.001**	1.72 [1.17-2.52]	**0.005**
high			Ref			
Income	Self-reported		Tested		Diagnosed	
	OR [95%CI]	p	OR [95%CI]	p	RR [95%CI]	p
Model 1						
low	0.86 [0.79-0.93]	**<0.001**	0.86 [0.78-0.95]	**0.002**	0.74 [0.47-1.18]	0.2
middle	0.80 [0.74-0.87]	**<0.001**	0.86 [0.78-0.94]	**0.002**	1.05 [0.70-1.57]	0.8
high			Ref			
Model 2						

low	0.85 [0.78-0.92]	**<0.001**	0.84 [0.76-0.92]	**<0.001**	0.78 [0.49-1.24]	0.3
middle	0.80 [0.74-0.86]	**<0.001**	0.85 [0.77-0.93]	**0.001**	1.04 [0.69-1.55]	**0.9**
high			Ref			

*Model 1: adjusted for education or income, age, sex, six lifestyle factors (smoking status, TV watching time ≥ 4h/day, sleep time <7 or >9 h/day, MVPA<150 min/week, LLDS, and heavy drinker), BMI, and ethnicity. Model 2: adjusted for education or income, age, sex, six lifestyle factors (smoking status, TV watching time ≥ 4h/day, sleep time <7 or >9 h/day, MVPA<150 min/week, LLDS, and heavy drinker), BMI, ethnicity, and number of questionnaires filled.

Supplementary Table 2. Characteristics and COVID-19 status of study population and excluded population.

	Study population n= 49,474	Excluded population n= 24,794
Sex, male %	40.3	37.3
Age, mean (sd)	46 ± 12	45 ± 12
BMI, mean (sd)	26.0 ± 4.2	26.0 ± 4.3
Underweight,%	0.7	0.8
Normal,%	44.9	42.0
Overweight,%	40.0	36.9
Obese,%	14.5	14.0
Ethnicity, white %	92.0	81.3
Self-reported covid-19, n (%)	4711 (9.5)	2534 (10.2)
Tested covid-19, n (%)	2883 (5.8)	1284 (5.2)
Diagnosed covid-19, n (%)	123 (0.3)	50 (0.2)

Supplementary Table 3. Mediation effect of lifestyle factors in the association between SES and COVID-19 status.

**SES indicators**	COVID-19 Status		
	Self-reported	Tested	Diagnosed
Education			
Proportion mediated (%)*	9.9	ns	ns
Income			
Proportion mediated (%)	6.7	9.5	ns

*The proportion mediated was calculated when the mediation effect of lifestyle factors was significant. As shown in the table, lifestyle factors only slightly mediated the association of SES and self-reported, and tested COVID-19 status because the proportion mediated was really small (<10%). This was in accordance with our regression model2 that the coefficient was slighted attenuated after adjustment for lifestyle factors.

Supplementary Table 4. COVID-19 diagnosed cases for the Netherlands as whole and for the Northern Provinces (Lifelines population). Source: RIVM, downloaded from the CoronaWatchNL Github.

Region	Diagnosed (cummulative on 28th of June, 2020)
Netherlands	50,167
North NL	1529
Groningen	362
Frisland	634
Drenthe	533
