# Supplementary material for: Association between socioeconomic status and self-reported, tested and diagnosed COVID-19 status during the first wave in the Northern Netherlands: a general population-based cohort from 49 474 adults
Source: BMJ Open. 2021 Mar 22;11(3):e048020. doi: 10.1136/bmjopen-2020-048020 (PMC7985974; doi:10.1136/bmjopen-2020-048020)
Supplement: Author's manuscript [file bmjopen-2020-048020.draft_revisions.pdf]

BMJ Open is committed to open peer review. As part of this commitment we make the peer review history of every article we publish publicly available.

When an article is published we post the peer reviewers' comments and the authors' responses online. We also post the versions of the paper that were used during peer review. These are the versions that the peer review comments apply to.

The versions of the paper that follow are the versions that were submitted during the peer review process. They are not the versions of record or the final published versions. They should not be cited or distributed as the published version of this manuscript.

BMJ Open is an open access journal and the full, final, typeset and author-corrected version of record of the manuscript is available on our site with no access controls, subscription charges or pay-per-view fees (http://bmjopen.bmj.com).

If you have any questions on BMJ Open's open peer review process please email info.bmjopen@bmj.com

BMJ Open

Socio-economic disparities in self-reported, tested, and diagnosed COVID-19: A general population-based cohort from 49,474 adults during the first wave in the Northern Netherlands

Journal:	*BMJ Open*
Manuscript ID	bmjopen-2020-048020
Article Type:	Original research
Date Submitted by the Author:	17-Dec-2020

Complete List of Authors:	Zhu, Yinjie; University Medical Centre Groningen,<br>Duan, Mingjie; University Medical Centre Groningen<br>Dijk, Hermien; University of Groningen Faculty of Economics and Business<br>Freriks, Roel; University of Groningen Faculty of Economics and Business<br>Dekker, Louise ; University Medical Centre Groningen, Nephrology<br>Mierau, Jochen; Rijksuniversiteit Groningen Faculteit Economie en Bedrijfskunde, Economics, Econometrics and Finance

Keywords:	COVID-19, Public health < INFECTIOUS DISEASES, Epidemiology < INFECTIOUS DISEASES

SCHOLARONE™
Manuscripts

I, the Submitting Author has the right to grant and does grant on behalf of all authors of the Work (as defined in the below author licence), an exclusive licence and/or a non-exclusive licence for contributions from authors who are: i) UK Crown employees; ii) where BMJ has agreed a CC-BY licence shall apply, and/or iii) in accordance with the terms applicable for US Federal Government officers or employees acting as part of their official duties; on a worldwide, perpetual, irrevocable, royalty-free basis to BMJ Publishing Group Ltd ("BMJ") its licensees and where the relevant Journal is co-owned by BMJ to the co-owners of the Journal, to publish the Work in this journal and any other BMJ products and to exploit all rights, as set out in our licence.

The Submitting Author accepts and understands that any supply made under these terms is made by BMJ to the Submitting Author unless you are acting as an employee on behalf of your employer or a postgraduate student of an affiliated institution which is paying any applicable article publishing charge ("APC") for Open Access articles. Where the Submitting Author wishes to make the Work available on an Open Access basis (and intends to pay the relevant APC), the terms of reuse of such Open Access shall be governed by a Creative Commons licence – details of these licences and which Creative Commons licence will apply to this Work are set out in our licence referred to above.

Other than as permitted in any relevant BMJ Author's Self Archiving Policies, I confirm this Work has not been accepted for publication elsewhere, is not being considered for publication elsewhere and does not duplicate material already published. I confirm all authors consent to publication of this Work and authorise the granting of this licence.

For peer review only

Title: Socio-economic disparities in self-reported, tested, and diagnosed COVID-19: A general population-based cohort from 49,474 adults during the first wave in the Northern Netherlands

Yinjie Zhu[1], Mingjie Duan[1], Hermien H. Dijk[2], Roel D. Freriks[2], Louise H. Dekker[1,3], Jochen O. Mierau[2,3]

[1] Department of Internal Medicine, Division of Nephrology, University of Groningen, University Medical Center Groningen, 9713 GZ Groningen, The Netherlands; [2] Faculty of Economics and Business, University of Groningen, 9747 AJ Groningen, The Netherlands; [3] Aletta Jacobs School of Public Health, University of Groningen, 9700 AS Groningen, The Netherlands.

Word count: Abstract, 237 words; Text, 2727 words; Tables, 3; Reference 36.

Running title: Socio-economic disparities in COVID-19 status

Key words: coronavirus, lifestyle, education, income, population cohort

Correspondence: Yinjie Zhu, y.zhu@umcg.nl

For peer review only

Abstract

Objectives

Studies in clinical settings showed a potential relationship between Socio-Economic Status (SES) and lifestyle factors with COVID-19, but it is still unknown whether this holds in the general population. In this study we investigated the associations of SES with self-reported, tested, and diagnosed COVID-19 status in the general population.

Design, setting, participants and outcome measures

Participants were 49,474 men and women (46 ± 12 yrs) residing in the Northern Netherlands from the Lifelines cohort study. SES indicators and lifestyle factors (i.e., smoking status, physical activity, alcohol intake, diet quality, sleep time, and TV watching time) were assessed by questionnaire from the Lifelines Biobank. Self-reported, tested, and diagnosed COVID-19 status were obtained from the Lifelines COVID-19 questionnaire.

Results

There were 4,711 participants who self-reported having had a COVID-19 infection, 2,883 participants tested for COVID-19, and 123 positive cases diagnosed in this study population. After adjustment for age, sex, lifestyle factors, BMI, and ethnicity, we found that participants with low education or low income were less likely to self-report a COVID-19 infection (OR [95%CI]: low education 0.78 [0.71-0.86]; low income 0.86 [0.79-0.93]), and be tested for COVID-19 (OR [95%CI]: low education 0.58 [0.52-0.66]; low income 0.86 [0.78-0.95]) compared with high education or high income groups, respectively.

Conclusion

Our findings suggest that the low SES group was the most vulnerable population to COVID-19 infection and self-reported and tested COVID-19 status in the general population was better predicted by SES than by lifestyle factors.

For peer review only

Strengths and limitations of this study

- This study added evidence to the socio-economically patterned COVID-19 status in a general population instead of in clinical settings.
- This study innovatively included a broader range of COVID-19 status, including self-reported and tested COVID-19 status, to better understand COVID-19 related socio-economic factors.
- This study might fail to identify the ethical differences because the study population was predominantly white (more than 98%).
- During the first wave of the COVID-19 pandemic in the Northern Netherlands, the number of cases identified were relatively low compared with the rest of the country, which might limit the power of the analysis about diagnosed COVID-19 status.

For peer review only - http://bmjopen.bmj.com/site/about/guidelines.xhtml

Introduction

The ongoing COVID-19 pandemic has already infected 62,363,527 people worldwide (as of December 1, 2020) [1]. Many studies have reported a relationship between lifestyle factors (e.g., smoking [2] and unhealthy diets [3]) or comorbidities associated with lifestyle factors (e.g., obesity [4] and diabetes mellitus [5]), and worse outcomes for COVID-19. Since lifestyle factors are often associated with Socio-Economic Status (SES) [6], and several studies have revealed that people with low SES are more susceptible to COVID-19 infection, hospitalization, and mortality [7-11], the interplay between SES, lifestyle and COVID-19 status becomes a valid query and it is likely that SES is a determinant of COVID-19.

Therefore, a rising concern among policy makers and public health practitioners is that the COVID-19 pandemic might exacerbate the persistent socio-economically patterned health inequalities over the life course [12, 13]. Still, an in-depth investigation of the lifestyle and socio-economic determinants of COVID-19 in the general population is lacking because current COVID-19 studies mostly relied on individuals presenting with symptoms in a clinical setting and focused on post COVID-19 infection status (e.g., hospitalization and intensive care) [14, 15]. As such, we lack knowledge on the validity of these early results for the general population.

This study aimed to investigate the extent to which SES and lifestyle factors were associated with a broader range of COVID-19 status, i.e. self-reported COVID-19, tested COVID-19, and diagnosed COVID-19 in the general population. To assess COVID-19 status in the general population we used data from the Lifelines COVID-19 Cohort. The Lifelines COVID-19 Cohort is a questionnaire-based study in a part of the Dutch Lifelines cohort [16]. To study the relationship between COVID-19 and SES, we linked data from the Lifelines COVID-19 questionnaire with data from the general Lifelines cohort, which contains a wide range of background data, such as demographics, lifestyles, and SES [17, 18]. As such, the findings of this study are less likely to be biased because of the population selection in clinical settings and can allow us to thoroughly understand the relation between SES and COVID-19 in the general population.

For peer review only - http://bmjopen.bmj.com/site/about/guidelines.xhtml

Methods

Study design and participants

For the present study, 49,474 participants from both the Lifelines cohort and the Lifelines COVID-19 Cohort, who had available and reliable data on demographics, SES, and lifestyle were included in the analysis (supplementary figure 1).

The Lifelines cohort study is a multidisciplinary prospective population-based cohort study based on a unique three-generation design. It employs a broad range of investigative procedures in assessing the biomedical, socio-demographic, behavioral, physical, and psychological factors which contribute to health and disease of the general population from the Northern Netherlands. A detailed description of the Lifelines cohort study can be found elsewhere [17, 18]. Before study entry, a signed informed consent form was obtained from each participant. Adult participants (≥ 18 years) were asked to complete several questionnaires regarding various aspects, including demographics, socio-economic status, and lifestyle. The Lifelines study is conducted according to the principles of the Declaration of Helsinki and approved by the Medical Ethics Committee of the University Medical Center Groningen, The Netherlands (2007/152).

The Lifelines COVID-19 Cohort collected data about COVID-19-related symptoms, current health issue and societal impacts from participants recruited from the Lifelines cohort. It is developed based on a COVID-19 questionnaire to identify genetic and environmental risk factors for COVID-19 and address the medical, social and psychological aspects of the pandemic. A detailed description of the Lifelines COVID-19 Cohort and the COVID-19 questionnaire can be found elsewhere [16]. The COVID-19 questionnaire is sent out weekly since March 30, 2020 for 12 weeks. Of the 139,713 Lifelines participants invited, 74,268 (53.2%) completed at least one of the questionnaires in the 12-week program.

COVID-19 Status

For peer review only - http://bmjopen.bmj.com/site/about/guidelines.xhtml

There were three different COVID-19 status obtained in our study from the COVID-19 questionnaire: self-reported COVID-19, tested COVID-19, and diagnosed COVID-19. All COVID-19 status was coded as binary variables. Self-reported COVID-19 status was obtained by asking “if you must choose, do you think you have (or have had) a COVID-19 infection?” or “has a doctor told you that you probably have (had) a coronavirus/COVID-19 infection”; tested COVID-19 status was obtained from the question “have you been tested for coronavirus (COVID-19)?”; diagnosed COVID-19 status was defined by asking “do you have or have you had a coronavirus/covid-19 infection?” or “what was the results of your corona virus (COVID-19) test?” when they had a COVID-19 test.

Since some of the COVID-related questions were worded such that it is not possible to infer whether the answer referred to the period between the last questionnaire and the current questionnaire, or to the beginning of the pandemic and the current questionnaire (e.g., “have you been tested for coronavirus (COVID-19)?”), the 12 questionnaires were condensed into a single observation per individual, indicating for each question whether the individual had at any point answered ‘yes’ during the study period.

Socio-Economic Status

Socio-Economic Status was indicated by a) highest educational level achieved and b) monthly net household income level separately from the Lifelines cohort study at baseline. Highest educational level achieved was categorized as: (1) low - junior general secondary education or lower (International Standard Classification of Education (ISCED) level 0, 1 or 2); (2) middle - secondary vocational education and senior general secondary education (ISCED level 3 or 4); and (3) high - higher vocational education or university (ISCED level 5 or 6) [19]; household net income level was categorized as: (1) low: <2000 euro/month; (2) middle: 2000-3000 euro/month; and (3) high: >3000 euro/month.

Lifestyle factors

Six lifestyle factors (i.e., smoking status, alcohol consumption, diet quality, physical activity, TV watching time, and sleep time) were selected from the Lifelines cohort study at baseline. Smoking

For peer review only - http://bmjopen.bmj.com/site/about/guidelines.xhtml

status was categorized into never, former, and current smoker. Alcohol intake and dietary consumption were derived from a validated 110-item semi-quantitative food frequency questionnaire (FFQ) that assessed food consumption over the past month [20]. Heavy drinking was defined as >40 g or >20 g average per day alcohol consumption for men and women, respectively [21]. Lifelines Diet Score (LLDS) was calculated to assess the overall diet quality based on the FFQ. This score ranks the relative intake of nine food groups with positive health effects (vegetables, fruit, whole grain products, legumes/nuts, fish, oils/soft margarines, unsweetened dairy, coffee, and tea) and three food groups with negative health effects (red/processed meat, butter/hard margarines, and sugar-sweetened beverages). The development of this score is described in detail elsewhere [22]. Non-occupational moderate-to-vigorous physical activity (MVPA) was calculated in minutes per week from the validated Short Questionnaire to ASsess Health-enhancing physical activity (SQUASH) data, which incorporated leisure time and commuting physical activities, including sports, at moderate (4.0-6.4 MET) to vigorous (≥6.5 MET) intensity [23]. TV watching time and sleep time were self-reported. Body mass index (BMI) was calculated by dividing weight in kilograms by the square of height in meters. The BMI was additionally categorized into underweight (BMI < 18.5 kg/m^2), normal (18.5 ≤ BMI < 25 kg/m^2), overweight (25 ≤ BMI < 30 kg/m^2), and obese (BMI ≥ 30 kg/m^2) [24].

Statistical analysis

Nominal variables are presented as percentage (%). Continuous variables were shown as mean ± standard deviation (SD) or median (interquartile range [IQR]). P-values < 0.05 were considered statistically significant. To analyze the associations of SES (i.e., education and income) with self-reported and tested COVID-19 status we used logistic regression models to estimate odds ratios (OR). Furthermore, we fitted robust Poisson regression models to estimate relative risk (RR) for associations between SES and diagnosed COVID-19 since the number of individuals with diagnosed COVID-19 was relatively low in this population. For all regression models, SES indicators were first entered into the model plus age and sex (model 1), and then adjusted for potential confounders (model 2: model 1 plus six lifestyle factors; model 3: model 2 plus BMI; model 4: model 3 plus ethnicity). All statistical analyses were conducted using Stata, version 13.1 (StataCorp, Texas, USA). Sensitivity analyses were

For peer review only - http://bmjopen.bmj.com/site/about/guidelines.xhtml

conducted by further adjusting for the total number of questionnaires filled by each participants (supplementary table 2), and by compare the study population and excluded participants have similar characteristics (supplementary table 3).

Patient and public involvement Patients and/or the public were not involved in the design, or conduct, or reporting, or dissemination plans of this research.

Results

Participant characteristics are shown in Table 1. The average age of the study population was 46 ± 12 years old and more women were recruited in this study (59.7%). More than 92% of the population characterized their ethnicity as white. The prevalence of overweight and obese were 40.0% and 14.5% respectively (Table 1). Out of the 49,474 participants that were recruited in both Lifelines cohort and Lifelines COVID-19 cohort, 4,711 participants self-reported a COVID-19 infection, while 2,833 participants reported having had an COVID-19 test, and 123 participants reported having had a positive outcome of the COVID-19 test (Table 1). Self-reported, tested, and diagnosed COVID-19 participants were more likely to be female, to have attained middle or high education, to have high income, and to have never smoked (Table 1).

The association between education and different COVID-19 status are presented in Table 2. Participants with low and middle education level were less likely to self-report COVID-19 (OR [95% CI]: low 0.78 [0.71-0.86]; middle 0.90 [0.84-0.97]), and be tested for COVID-19 (OR [95% CI]: low 0.58 [0.52-0.66]; middle 0.72 [0.66-0.79]) compared with highly educated participants after adjustment for age, sex, lifestyle factors, ethnicity, and BMI (Table 2). In addition, compared with high education participants, participants with middle education level had almost two-fold higher risk to be infected with COVID-19 (OR [95%]: 1.73 [1.19-2.50]) (Table 2, model 1) once they had been tested for COVID-19. The association was slightly attenuated after adjustment for lifestyle factors (OR [95%]: 1.69 [1.17-2.44]) (Table 2, model 2), however, further adjustment for BMI and ethnicity did not change the results.

For peer review only - http://bmjopen.bmj.com/site/about/guidelines.xhtml

For peer review only

The association between income and different COVID-19 status are presented in Table 3. Low or middle income groups were less likely to self-report COVID-19 (OR [95% CI]: low 0.86 [0.79-0.93]; middle 0.80 [0.74-0.87]), and to be tested for COVID-19 (OR [95% CI]: low 0.86 [0.78-0.95]; middle 0.86 [0.78-0.94]) compared with high income group after adjustment for all covariates (table 3, model 4). No risk differences were found between different income levels and diagnosed COVID-19 status, once tested.

Discussion

This paper is the first to provide insight in socio-economic disparities on self-reported COVID-19, tested COVID-19, and diagnosed COVID-19 in the general population. We found that low and middle SES groups, especially participants with low education, were less likely to self-report COVID-19 and to be tested for COVID-19. Moreover, participants with middle education were more likely to be infected with COVID-19 compared with those who obtained high education.

Our study highlighted the importance of mitigating the socio-economically patterned effect of the COVID-19 pandemic by showing a clear relationship between SES and COVID-19 status, which enables and aids policy makers to adequately respond to the pandemic and develop public health measurements. It is worrisome that low SES groups are likely to be neglected or undetected during the pandemic because they are less likely to self-report and be tested for COVID-19. Given the fact that they are also at high risk of non-communicable diseases [25], once infected with COVID-19 or other infectious disease in the future, individuals with low SES might suffer disproportionately from the disease development compared with the high SES group, which may further exacerbate health inequality [26]. Therefore, national or regional commitment of public health measures, guidelines, or interventions are needed to promote health equity before, during, and after COVID-19 pandemic by identifying, targeting, and engaging the low SES group.

One of the potential mechanisms is poor health literacy. Health literacy refers to “a person’s knowledge, motivation, and competences to access, understand , appraise, and apply health

For peer review only

For peer review only - http://bmjopen.bmj.com/site/about/guidelines.xhtml

information in order to make judgments and take decisions in everyday life" [27]. It is known that low SES is the most important determinant of health literacy [28]. During the COVID-19 pandemic, people with high SES may be more aware of the symptoms and health consequences of COVID-19, and can, therefore, better interpret and communicate and make a decision about their own health condition [29, 30]. In other words, low SES people might be less sensitive and aware of their COVID-19 related symptoms, or they might lack the ability to assess if they could have had COVID-19 infection. Since they might lack health literacy, low SES people might be less likely to seek medical help even when they have symptoms because of the lack of practicing health seeking behavior and critical health resources [12, 13]. On the other hand, potentially low SES individuals had more difficulties obtaining a test even when they had sufficient health literacy. Our results also suggested that education level is a better determinant for COVID-19 status than income [28] because the association gradient was larger for low education participants than for low income participants. Further research is needed to fully understand the observed link between socio-economic disparities and COVID-19.

Interestingly, lifestyle factors seemed not to be a mediator in the pathway between SES and diagnosed COVID-19 status in our study, which is different from a previous study that suggested an unhealthy lifestyle was a profound risk factor for COVID-19 status [15]. Some other studies also found that nutritional status [31, 32] and smoking [2] might also be risk factors for COVID-19 disease severity, disease progression, and mortality. Because of the differences of study population and COVID-19 outcome selection, our results were not comparable with previous studies. Nevertheless, this study provided a fresh message that in the general population, SES is a better determinant of self-reported, tested, and diagnosed COVID-19 status. In addition, unlike other COVID-19 studies that found obesity and ethnicity were both risk factors of COVID-19 infection, hospitalization, and mortality [10, 11, 15, 33-36], our results showed that obesity and ethnicity did not contribute substantially to the disparities in COVID-19 status, which indicates that obesity and ethnicity were not in the pathway between SES and self-reported, tested, and diagnosed COVID-19 status.

For peer review only - http://bmjopen.bmj.com/site/about/guidelines.xhtml

This study has several strengths. First, this general population-based study about COVID-19 may to some extent mitigate the selection bias compared with studies conducted based in clinical settings. Second, we innovatively included a broader range of COVID-19 status, including self-reported and tested COVID-19 status, to better understand COVID-19 related health literacy and accessibility to health services. A major limitation of the current study is the fact that the study population was predominantly white (more than 98%). Additionally, the Netherlands has a well-developed social security system. This may limit its generalizability to populations of other ethnicity, and in a different social context. One caveat of our study is that the relative low number of cases in the region, which is the nature of the COVID-19 outbreak in the Northern Netherlands (supplementary table 1), might influence the statistical power of the study. However, we still find statistically significant results for SES and COVID-19 status. Another limitation is from the condensed observations about COVID-19 status since certain types of individuals might be more likely to fill in more questionnaires, and are therefore more likely to report a positive COVID-19 status in at least one of the questionnaires. If these individuals predominantly belong to a specific SES group, this would bias the results. However, by further adjusting for the total number of questionnaires filled by each participants, we did not observe any difference from the current results (supplementary table 2). Lastly, approximately 17% of the participants were dropped because of missing SES data, while 16% participants were dropped because of incomplete lifestyle information, which might bias the results given that people with low SES and worse lifestyle practices could be less likely to participant in the COVID-19 questionnaire. Nevertheless, both the study population and excluded participants have similar characteristics (supplementary table 3). Consequently, it is less likely that there was a selection bias resulting from missing variables.

In conclusion, our findings indicate that individuals with lower SES may be more vulnerable during COVID-19 pandemic, suggesting that health services and health promotion interventions should particularly focus on targeting individuals with lower SES to get them better prepared for future health emergencies.

For peer review only - http://bmjopen.bmj.com/site/about/guidelines.xhtml

Contributions YZ: analysis design and execution, drafted the manuscript. MD: literature review, study design, manuscript review and approval. HD: study design, supervision, manuscript review and approval. RDF: study design, supervision, manuscript review and approval. LHD: conception, supervision, manuscript review and approval. JOM: conception, manuscript review and approval, stakeholder engagement.

Acknowledgement The authors wish to acknowledge the services of the Lifelines cohort study, the contributing research centers delivering data to Lifelines, and all the study participants.

Funding This project has received funding from the European Union's Horizon 2020 research and innovation programme under the Marie Skłodowska-Curie grant agreement No 754425 as well as funding from NWO-Fast Track No 440.20.002. The Lifelines Biobank initiative has been made possible by funds from FES (Fonds Economische Structuurversterking), SNN (Samenwerkingsverband Noord Nederland) and REP (Ruimtelijk Economisch Programma).

Competing interests The authors have declared that no competing interests exist.

Patient and public involvement Patients and/or the public were not involved in the design, or conduct, or reporting, or dissemination plans of this research.

Ethics approval The Lifelines study is conducted according to the principles of the Declaration of Helsinki and approved by the Medical Ethics Committee of the University Medical Center Groningen, The Netherlands (2007/152). Before study entry, a signed informed consent form was obtained from each participant.

Data availability statement Data may be obtained from a third party and are not publicly available.

For peer review only

Reference

1. WHO. *WHO Coronavirus Disease (COVID-19) Dashboard*. 2020; Available from: https://covid19.who.int/?gclid=EAIaIQobChMIzsuel8uQ7AIVgrh3Ch2FbgICEAAYASAAEgL5y_D_BwE.
2. Vardavas, C.I. and K. Nikitara, *COVID-19 and smoking: A systematic review of the evidence.* Tobacco Induced Diseases, 2020. **18**(March).
3. Butler, M.J. and R.M. Barrientos, *The impact of nutrition on COVID-19 susceptibility and long-term consequences.* Brain, Behavior, and Immunity, 2020. **87**: p. 53-54.
4. Tartof, S.Y., et al., *Obesity and Mortality Among Patients Diagnosed With COVID-19: Results From an Integrated Health Care Organization.* Ann Intern Med, 2020.
5. Cuschieri, S. and S. Grech, *COVID-19 and diabetes: The why, the what and the how.* Journal of Diabetes and its Complications, 2020. **34**(9): p. 107637.
6. Glorioso, V. and M. Pisati, *Socioeconomic inequality in health-related behaviors: a lifestyle approach.* Quality & Quantity, 2014. **48**(5): p. 2859-2879.
7. Batty, G.D., et al., *Psychosocial factors and hospitalisations for COVID-19: Prospective cohort study based on a community sample.* Brain Behav Immun, 2020.
8. McCarthy, C.P., et al., *Early clinical and sociodemographic experience with patients hospitalized with COVID-19 at a large American healthcare system.* EClinicalMedicine, 2020: p. 100504.
9. Khalatbari-Soltani, S., et al., *Importance of collecting data on socioeconomic determinants from the early stage of the COVID-19 outbreak onwards.* J Epidemiol Community Health, 2020. **74**(8): p. 620-623.
10. Abedi, V., et al., *Racial, Economic and Health Inequality and COVID-19 Infection in the United States.* medRxiv, 2020.
11. Wiemers, E.E., et al., *Disparities in Vulnerability to Severe Complications from COVID-19 in the United States.* medRxiv, 2020.
12. Burström, B. and W. Tao, *Social determinants of health and inequalities in COVID-19.* European Journal of Public Health, 2020. **30**(4): p. 617-618.
13. Schulz, A.J., et al., *Moving Health Education and Behavior Upstream: Lessons From COVID-19 for Addressing Structural Drivers of Health Inequities.* Health Educ Behav, 2020. **47**(4): p. 519-524.
14. Wynants, L., et al., *Prediction models for diagnosis and prognosis of covid-19: systematic review and critical appraisal.* BMJ, 2020. **369**: p. m1328.
15. Hamer, M., et al., *Lifestyle risk factors, inflammatory mechanisms, and COVID-19 hospitalization: A community-based cohort study of 387,109 adults in UK.* Brain, Behavior, and Immunity, 2020. **87**: p. 184-187.
16. Mc Intyre, K., et al., *The Lifelines COVID-19 Cohort: a questionnaire-based study to investigate COVID-19 infection and its health and societal impacts in a Dutch population-based cohort.* medRxiv, 2020: p. 2020.06.19.20135426.
17. Klijs, B., et al., *Representativeness of the LifeLines Cohort Study.* PLoS One, 2015. **10**(9): p. e0137203.
18. Scholtens, S., et al., *Cohort Profile: LifeLines, a three-generation cohort study and biobank.* International Journal of Epidemiology, 2014. **44**(4): p. 1172-1180.
19. *International standard classification of education*. 1997, United nations educational, scientific, and cultural organization.
20. Siebelink, E., A. Geelen, and J.H. de Vries, *Self-reported energy intake by FFQ compared with actual energy intake to maintain body weight in 516 adults.* Br J Nutr, 2011. **106**(2): p. 274-81.
21. Dawson, D.A., *Defining risk drinking.* Alcohol research & health : the journal of the National Institute on Alcohol Abuse and Alcoholism, 2011. **34**(2): p. 144-156.
22. Vinke, P.C., et al., *Development of the food-based Lifelines Diet Score (LLDS) and its application in 129,369 Lifelines participants.* Eur J Clin Nutr, 2018. **72**(8): p. 1111-1119.

For peer review only - http://bmjopen.bmj.com/site/about/guidelines.xhtml

23. Wendel-Vos, G.C.W., et al., *Reproducibility and relative validity of the short questionnaire to assess health-enhancing physical activity.* Journal of Clinical Epidemiology, 2003. **56**(12): p. 1163-1169.
24. WHO, *Obesity : preventing and managing the global epidemic : report of a WHO consultation*, in *WHO Technical Report Series*. 1999, World Health Organization: Geneva, Switzerland.
25. Collaborators, G.B.D.C.o.D., *Global, regional, and national age-sex-specific mortality for 282 causes of death in 195 countries and territories, 1980-2017: a systematic analysis for the Global Burden of Disease Study 2017.* Lancet (London, England), 2018. **392**(10159): p. 1736-1788.
26. Marmot, M. and J. Allen, *COVID-19: exposing and amplifying inequalities.* Journal of Epidemiology and Community Health, 2020. **74**(9): p. 681.
27. Sørensen, K., et al., *Health literacy and public health: A systematic review and integration of definitions and models.* BMC Public Health, 2012. **12**(1): p. 80.
28. Stormacq, C., S. Van den Broucke, and J. Wosinski, *Does health literacy mediate the relationship between socioeconomic status and health disparities? Integrative review.* Health Promotion International, 2019. **34**(5): p. e1-e17.
29. Zhong, B.L., et al., *Knowledge, attitudes, and practices towards COVID-19 among Chinese residents during the rapid rise period of the COVID-19 outbreak: a quick online cross-sectional survey.* Int J Biol Sci, 2020. **16**(10): p. 1745-1752.
30. Alobuia, W.M., et al., *Racial disparities in knowledge, attitudes and practices related to COVID-19 in the USA.* J Public Health (Oxf), 2020. **42**(3): p. 470-478.
31. Richardson, D.P. and J.A. Lovegrove, *Nutritional status of micronutrients as a possible and modifiable risk factor for COVID-19: a UK perspective.* British Journal of Nutrition, 2020: p. 1-7.
32. Zabetakis, I., et al., *COVID-19: The Inflammation Link and the Role of Nutrition in Potential Mitigation.* Nutrients, 2020. **12**(5).
33. Sattar, N., et al., *BMI and future risk for COVID-19 infection and death across sex, age and ethnicity: Preliminary findings from UK biobank.* Diabetes & Metabolic Syndrome: Clinical Research & Reviews, 2020. **14**(5): p. 1149-1151.
34. Patel, A.P., et al., *Race, socioeconomic deprivation, and hospitalization for COVID-19 in English participants of a national biobank.* Int J Equity Health, 2020. **19**(1): p. 114.
35. Raisi-Estabragh, Z., et al., *Greater risk of severe COVID-19 in Black, Asian and Minority Ethnic populations is not explained by cardiometabolic, socioeconomic or behavioural factors, or by 25(OH)-vitamin D status: study of 1326 cases from the UK Biobank.* J Public Health (Oxf), 2020. **42**(3): p. 451-460.
36. Adhikari, S., et al., *Assessment of Community-Level Disparities in Coronavirus Disease 2019 (COVID-19) Infections and Deaths in Large US Metropolitan Areas.* JAMA network open, 2020. **3**(7): p. e2016938-e2016938.

For peer review only

For peer review only - http://bmjopen.bmj.com/site/about/guidelines.xhtml

Table 1. Demographics, socio-economic status, and lifestyle of the study population

	COVID-19 Status			
	Self-reported (n=4711)	Tested (2883)	Diagnosed (123)	Total response (n=49,474)
Sex, male%	37.4	30.4	30.1	40.3
Age, mean ± sd	42 ± 11	44 ± 12	44 ± 9	46 ± 12
Ethnicity, white%	89.4	92.8	94.3	92.0
BMI, mean ± sd	25.9 ± 4.3	25.7 ± 4.3	25.7 ± 3.8	26.0 ± 4.2
Underweight,%	0.7	1.0	0.8	0.7
Normal,%	46.7	48.0	47.2	44.9
Overweight,%	37.2	36.6	40.7	40.0
Obese,%	15.4	14.5	11.4	14.5
Education,%				
Low	18.0	16.4	14.6	23.5
Middle	40.8	37.4	48.0	39.2
High	41.2	46.2	37.4	37.3
Income, %				
Low	30.1	28.3	23.6	28.1
Middle	29.5	30.7	33.3	33.7
High	40.4	41.0	43.1	38.2
Smoking, %				
Current	20.5	15.1	15.5	16.9
Former	33.0	36.1	40.7	36.3
Never	46.5	48.9	43.9	46.8
TV watching time ≥ 4h/day,%	15.2	15.1	18.7	16.7
Sleep time <7 or >9 h/day, %	16.3	14.4	13.8	14.9
MVPA<150 min/week,%	38.8	37.5	29.3	37.9
LLDS	24.3 ± 6.1	25.1 ± 6.0	25.4 ± 5.9	24.7 ± 6.0
Alcohol, g/day	4.9 (1.3-11.0)	3.9 (0.9-10.0)	3.5 (0.9-9.0)	4.9 (1.2-11.1)
Heavy drinker, %	3.1	2.8	2.4	2.6

*Abbreviations: *sd* standard deviation; *LLDS* lifelines diet score; *MVPA* non-occupational moderate-to-vigorous physical activity

Table 2. Association of education with different COVID-19 status

Education	COVID-19 Status					
	Self-reported		Tested		Diagnosed	
	OR [95%CI]	p	OR [95%CI]	p	RR [95%CI]	p
Model 1						
low	0.81 [0.75-0.89]	**<0.001**	0.58 [0.52-0.65]	**<0.001**	1.18 [0.67-2.07]	0.6
middle	0.93 [0.87-0.99]	**0.03**	0.73 [0.67-0.80]	**<0.001**	1.73 [1.19-2.50]	**0.004**
high			Ref			
Model 2						
low	0.79 [0.72-0.86]	**<0.001**	0.59 [0.53-0.66]	**<0.001**	1.11 [0.61-2.00]	0.7
middle	0.91 [0.85-0.97]	**0.006**	0.74 [0.68-0.81]	**<0.001**	1.69 [1.17-2.44]	**0.005**
high			Ref			
Model 3						
low	0.77 [0.71-0.85]	**<0.001**	0.59 [0.53-0.66]	**<0.001**	1.11 [0.61-2.00]	0.7

For peer review only

For peer review only - http://bmjopen.bmj.com/site/about/guidelines.xhtml

middle	0.90 [0.84-0.96]	**0.002**	0.74 [0.68-0.81]	**<0.001**	1.69 [1.16-2.45]	**0.006**
high			Ref			
Model 4						
low	0.78 [0.71-0.86]	**<0.001**	0.58 [0.52-0.66]	**<0.001**	1.10 [0.60-2.01]	0.8
middle	0.90 [0.84-0.97]	**0.004**	0.72 [0.66-0.79]	**<0.001**	1.77 [1.21-2.60]	**0.003**
high			Ref			

*Model 1: adjusted for education, age and sex. Model 2: adjusted for education, age, sex, and six lifestyle factors (smoking status, TV watching time ≥ 4h/day, sleep time <7 or >9 h/day, MVPA<150 min/week, LLDS, and heavy drinker). Model3: adjusted for education, age, sex, six lifestyle factors (smoking status, TV watching time ≥ 4h/day, sleep time <7 or >9 h/day, MVPA<150 min/week, LLDS, and heavy drinker), and BMI. Model 4: adjusted for education, age, sex, six lifestyle factors (smoking status, TV watching time ≥ 4h/day, sleep time <7 or >9 h/day, MVPA<150 min/week, LLDS, and heavy drinker), BMI, and ethnicity.

Table 3. Association of income with different COVID-19 status

Income	COVID-19 Status					
	Self-reported		Tested		Diagnosed	
	OR [95%CI]	p	OR [95%CI]	p	RR [95%CI]	p
Model 1						
low	0.89 [0.83-0.96]	**0.002**	0.85 [0.77-0.93]	**0.001**	0.84 [0.54-1.31]	0.4
middle	0.82 [0.76-0.88]	**<0.001**	0.84 [0.77-0.92]	**<0.001**	1.14 [0.77-1.70]	0.5
high			Ref			
Model 2						
low	0.88 [0.81-0.95]	**0.001**	0.87 [0.79-0.96]	**0.005**	0.80 [0.51-1.25]	0.3
middle	0.82 [0.76-0.88]	**<0.001**	0.86 [0.78-0.94]	**0.001**	1.10 [0.73-1.64]	0.6
high			Ref			
Model 3						
low	0.88 [0.81-0.95]	**0.001**	0.87 [0.79-0.96]	**0.006**	0.80 [0.51-1.25]	0.3
middle	0.81 [0.76-0.87]	**<0.001**	0.86 [0.78-0.94]	**0.001**	1.10 [0.73-1.64]	0.7
high			Ref			
Model 4						
low	0.86 [0.79-0.93]	**<0.001**	0.86 [0.78-0.95]	**0.002**	0.74 [0.47-1.18]	0.2
middle	0.80 [0.74-0.87]	**<0.001**	0.86 [0.78-0.94]	**0.002**	1.05 [0.70-1.57]	0.8
high			Ref			

*Model 1: adjusted for income, age and sex. Model 2: adjusted for income, age, sex, and six lifestyle factors (smoking status, TV watching time ≥ 4h/day, sleep time <7 or >9 h/day, MVPA<150 min/week, LLDS, and heavy drinker). Model3: adjusted for income, age, sex, six lifestyle factors (smoking status, TV watching time ≥ 4h/day, sleep time <7 or >9 h/day, MVPA<150 min/week, LLDS, and heavy drinker), and BMI. Model 4: adjusted for income, age, sex, six lifestyle factors (smoking status, TV watching time ≥ 4h/day, sleep time <7 or >9 h/day, MVPA<150 min/week, LLDS, and heavy drinker), BMI, and ethnicity.

For peer review only

For peer review only - http://bmjopen.bmj.com/site/about/guidelines.xhtml

Supplementary Figure 1. Study flow chart

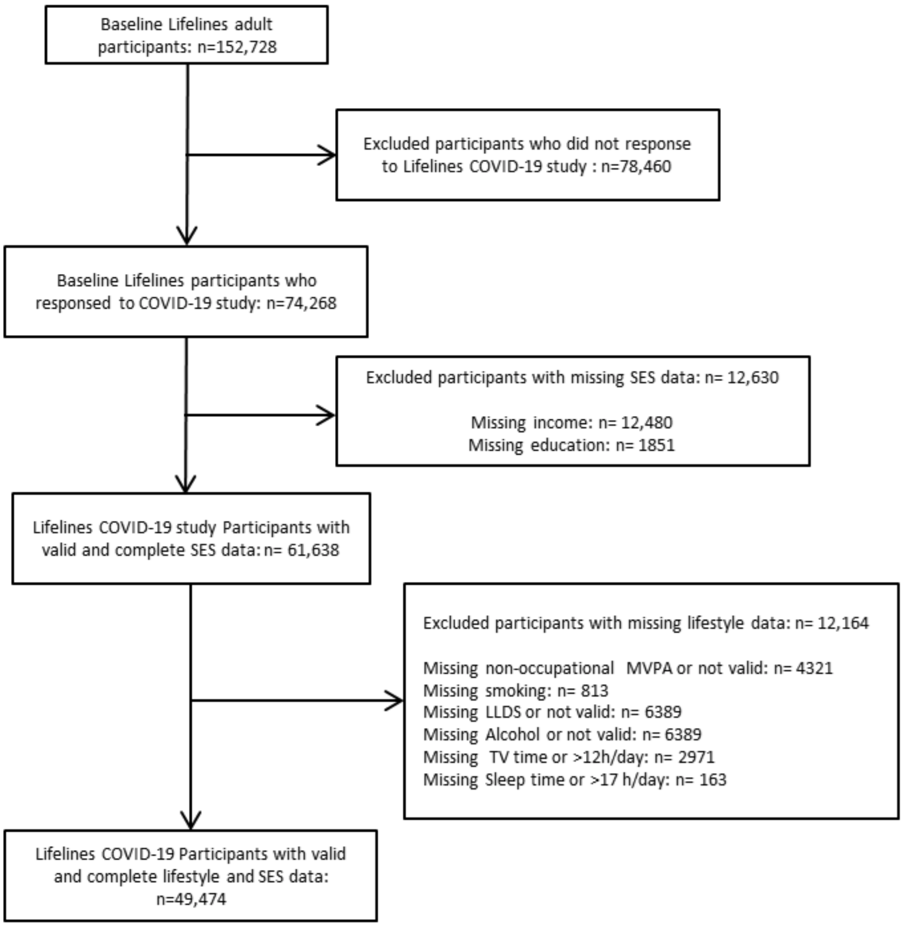

Supplementary Table 1. COVID-19 diagnosed cases for the Netherlands as whole and for the Northern Provinces (Lifelines population). Source: RIVM, downloaded from the CoronaWatchNL Github.

Region	Diagnosed (cummulative on 28th of June, 2020)
Netherlands	50,167
North NL	1529
Groningen	362
Frisland	634
Drenthe	533

Supplementary Table 2. Association of education or income with COVID-19 status with further adjustment for number of questionnaires filled.

Education	COVID-19 Status					
	Self-reported		Tested		Diagnosed	
	OR [95%CI]	p	OR [95%CI]	p	RR [95%CI]	p
Model 1						
low	0.78 [0.71-0.86]	**<0.001**	0.58 [0.52-0.66]	**<0.001**	1.10 [0.60-2.01]	0.8
middle	0.90 [0.84-0.97]	**0.004**	0.72 [0.66-0.79]	**<0.001**	1.77 [1.21-2.60]	**0.003**
high			Ref			
Model 2						

For peer review only

low	0.79 [0.72-0.87]	**<0.001**	0.61 [0.54-0.68]	**<0.001**	1.08 [0.60-1.95]	0.8
middle	0.91 [0.84-0.97]	**0.006**	0.73 [0.67-0.80]	**<0.001**	1.72 [1.17-2.52]	**0.005**
high			Ref			

Income	Self-reported		Tested		Diagnosed	
	OR [95%CI]	p	OR [95%CI]	p	RR [95%CI]	p
Model 1						
low	0.86 [0.79-0.93]	**<0.001**	0.86 [0.78-0.95]	**0.002**	0.74 [0.47-1.18]	0.2
middle	0.80 [0.74-0.87]	**<0.001**	0.86 [0.78-0.94]	**0.002**	1.05 [0.70-1.57]	0.8
high			Ref			
Model 2						
low	0.85 [0.78-0.92]	**<0.001**	0.84 [0.76-0.92]	**<0.001**	0.78 [0.49-1.24]	0.3
middle	0.80 [0.74-0.86]	**<0.001**	0.85 [0.77-0.93]	**0.001**	1.04 [0.69-1.55]	**0.9**
high			Ref			

*Model 1: adjusted for education or income, age, sex, six lifestyle factors (smoking status, TV watching time ≥ 4h/day, sleep time <7 or >9 h/day, MVPA<150 min/week, LLDS, and heavy drinker), BMI, and ethnicity. Model 2: adjusted for education or income, age, sex, six lifestyle factors (smoking status, TV watching time ≥ 4h/day, sleep time <7 or >9 h/day, MVPA<150 min/week, LLDS, and heavy drinker), BMI, ethnicity, and number of questionnaires filled.

Supplementary Table 3. Characteristics and COVID-19 status of study population and excluded population.

	Study population n= 49,474	Excluded population n= 24,794
Sex, male %	40.3	37.3
Age, mean (sd)	46 ± 12	45 ± 12
BMI, mean (sd)	26.0 ± 4.2	26.0 ± 4.3
Underweight,%	0.7	0.8
Normal,%	44.9	42.0
Overweight,%	40.0	36.9
Obese,%	14.5	14.0
Ethnicity, white %	92.0	81.3
Self-reported covid-19, n (%)	4711 (9.5)	2534 (10.2)
Tested covid-19, n (%)	2883 (5.8)	1284 (5.2)
Diagnosed covid-19, n (%)	123 (0.3)	50 (0.2)

For peer review only

STROBE Statement—checklist of items that should be included in reports of observational studies

	Item No	Recommendation	Page No
**Title and abstract**	1	(*a*) Indicate the study's design with a commonly used term in the title or the abstract	1
		(*b*) Provide in the abstract an informative and balanced summary of what was done and what was found	2
**Introduction**			
Background/rationale	2	Explain the scientific background and rationale for the investigation being reported	4
Objectives	3	State specific objectives, including any prespecified hypotheses	4
**Methods**			
Study design	4	Present key elements of study design early in the paper	5
Setting	5	Describe the setting, locations, and relevant dates, including periods of recruitment, exposure, follow-up, and data collection	5
Participants	6	(*a*) *Cohort study*—Give the eligibility criteria, and the sources and methods of selection of participants. Describe methods of follow-up<br>*Case-control study*—Give the eligibility criteria, and the sources and methods of case ascertainment and control selection. Give the rationale for the choice of cases and controls<br>*Cross-sectional study*—Give the eligibility criteria, and the sources and methods of selection of participants	5
		(*b*) *Cohort study*—For matched studies, give matching criteria and number of exposed and unexposed<br>*Case-control study*—For matched studies, give matching criteria and the number of controls per case	
Variables	7	Clearly define all outcomes, exposures, predictors, potential confounders, and effect modifiers. Give diagnostic criteria, if applicable	6-7
Data sources/ measurement	8*	For each variable of interest, give sources of data and details of methods of assessment (measurement). Describe comparability of assessment methods if there is more than one group	5-7
Bias	9	Describe any efforts to address potential sources of bias	10
Study size	10	Explain how the study size was arrived at	supplementary
Quantitative variables	11	Explain how quantitative variables were handled in the analyses. If applicable, describe which groupings were chosen and why	5-7
Statistical methods	12	(*a*) Describe all statistical methods, including those used to control for confounding	7
		(*b*) Describe any methods used to examine subgroups and interactions	
		(*c*) Explain how missing data were addressed	5
		(*d*) *Cohort study*—If applicable, explain how loss to follow-up was addressed<br>*Case-control study*—If applicable, explain how matching of cases and controls was addressed<br>*Cross-sectional study*—If applicable, describe analytical methods taking account of sampling strategy	

For peer review only - http://bmjopen.bmj.com/site/about/guidelines.xhtml

(*e*) Describe any sensitivity analyses	7-8

Continued on next page

For peer review only

For peer review only - http://bmjopen.bmj.com/site/about/guidelines.xhtml

**Results**			
Participants	13*	(a) Report numbers of individuals at each stage of study—eg numbers potentially eligible, examined for eligibility, confirmed eligible, included in the study, completing follow-up, and analysed	8
		(b) Give reasons for non-participation at each stage	
		(c) Consider use of a flow diagram	supplementary
Descriptive data	14*	(a) Give characteristics of study participants (eg demographic, clinical, social) and information on exposures and potential confounders	8
		(b) Indicate number of participants with missing data for each variable of interest	supplementary
		(c) *Cohort study*—Summarise follow-up time (eg, average and total amount)	
Outcome data	15*	*Cohort study*—Report numbers of outcome events or summary measures over time	
		Case-control study—Report numbers in each exposure category, or summary measures of exposure	
		Cross-sectional study—Report numbers of outcome events or summary measures	*8*
Main results	16	(*a*) Give unadjusted estimates and, if applicable, confounder-adjusted estimates and their precision (eg, 95% confidence interval). Make clear which confounders were adjusted for and why they were included	8-9
		(*b*) Report category boundaries when continuous variables were categorized	
		(*c*) If relevant, consider translating estimates of relative risk into absolute risk for a meaningful time period	
Other analyses	17	Report other analyses done—eg analyses of subgroups and interactions, and sensitivity analyses	11
**Discussion**			
Key results	18	Summarise key results with reference to study objectives	9
Limitations	19	Discuss limitations of the study, taking into account sources of potential bias or imprecision. Discuss both direction and magnitude of any potential bias	11
Interpretation	20	Give a cautious overall interpretation of results considering objectives, limitations, multiplicity of analyses, results from similar studies, and other relevant evidence	10
Generalisability	21	Discuss the generalisability (external validity) of the study results	9
**Other information**			
Funding	22	Give the source of funding and the role of the funders for the present study and, if applicable, for the original study on which the present article is based	12

*Give information separately for cases and controls in case-control studies and, if applicable, for exposed and unexposed groups in cohort and cross-sectional studies.

Note: An Explanation and Elaboration article discusses each checklist item and gives methodological background and published examples of transparent reporting. The STROBE checklist is best used in conjunction with this article (freely available on the Web sites of PLoS Medicine at http://www.plosmedicine.org/, Annals of Internal Medicine at http://www.annals.org/, and Epidemiology at http://www.epidem.com/). Information on the STROBE Initiative is available at www.strobe-statement.org.

For peer review only

For peer review only - http://bmjopen.bmj.com/site/about/guidelines.xhtml

Association between socio-economic status and self-reported, tested, and diagnosed COVID-19 status during the first wave in the Northern Netherlands: A general population-based cohort from 49,474 adults

Journal:	*BMJ Open*
Manuscript ID	bmjopen-2020-048020.R1
Article Type:	Original research
Date Submitted by the Author:	13-Feb-2021
Complete List of Authors:	Zhu, Yinjie; University Medical Centre Groningen,<br>Duan, Mingjie; University Medical Centre Groningen<br>Dijk, Hermien; University of Groningen Faculty of Economics and Business<br>Freriks, Roel; University of Groningen Faculty of Economics and Business<br>Dekker, Louise ; University Medical Centre Groningen, Nephrology<br>Mierau, Jochen; Rijksuniversiteit Groningen Faculteit Economie en Bedrijfskunde, Economics, Econometrics and Finance
**Primary Subject Heading**:	Epidemiology
Secondary Subject Heading:	Public health, Infectious diseases, Health policy
Keywords:	COVID-19, Public health < INFECTIOUS DISEASES, Epidemiology < INFECTIOUS DISEASES

I, the Submitting Author has the right to grant and does grant on behalf of all authors of the Work (as defined in the below author licence), an exclusive licence and/or a non-exclusive licence for contributions from authors who are: i) UK Crown employees; ii) where BMJ has agreed a CC-BY licence shall apply, and/or iii) in accordance with the terms applicable for US Federal Government officers or employees acting as part of their official duties; on a worldwide, perpetual, irrevocable, royalty-free basis to BMJ Publishing Group Ltd ("BMJ") its licensees and where the relevant Journal is co-owned by BMJ to the co-owners of the Journal, to publish the Work in this journal and any other BMJ products and to exploit all rights, as set out in our licence.

The Submitting Author accepts and understands that any supply made under these terms is made by BMJ to the Submitting Author unless you are acting as an employee on behalf of your employer or a postgraduate student of an affiliated institution which is paying any applicable article publishing charge ("APC") for Open Access articles. Where the Submitting Author wishes to make the Work available on an Open Access basis (and intends to pay the relevant APC), the terms of reuse of such Open Access shall be governed by a Creative Commons licence – details of these licences and which Creative Commons licence will apply to this Work are set out in our licence referred to above.

Other than as permitted in any relevant BMJ Author's Self Archiving Policies, I confirm this Work has not been accepted for publication elsewhere, is not being considered for publication elsewhere and does not duplicate material already published. I confirm all authors consent to publication of this Work and authorise the granting of this licence.

For peer review only

Title: Association between socio-economic status and self-reported, tested, and diagnosed COVID-19 status during the first wave in the Northern Netherlands: A general population-based cohort from 49,474 adults

Yinjie Zhu[1], Ming-Jie Duan[1], Hermien H. Dijk[2], Roel D. Freriks[2], , Louise H. Dekker[1,3], Jochen O. Mierau[2,3], Lifelines Corona Research initiative[2,3,4*]

[1] Department of Internal Medicine, Division of Nephrology, University of Groningen, University Medical Center Groningen, Hanzeplein 1, 9713 GZ Groningen, The Netherlands; [2] Faculty of Economics and Business, University of Groningen, Universiteitscomplex, 9747 AJ Groningen, The Netherlands; [3] Aletta Jacobs School of Public Health, University of Groningen, Oude Boteringestraat 24, 9712 GH Groningen, The Netherlands; [4] Faculty of Medical Science, University Medical Center Groningen, Hanzeplein 1, 9713GZ Groningen, The Netherlands.

Word count: Abstract, 230 words; Text, 3088 words; Tables, 3; Reference 38.

Running title: Socio-economic disparities in COVID-19 status

Key words: coronavirus, lifestyle, education, income, population cohort

Correspondence: Yinjie Zhu, y.zhu@umcg.nl

For peer review only

*Lifelines Corona Research initiative: Marike Boezen; Jochen Mierau; Lude Franke; Jackie Dekens; Patrick Deelen; Pauline Lanting; Judith Vonk; Ilja Nolte; Anil Ori; Annique Claringbould; Floranne Boulogne; Marjolein Dijkema; Henry Wiersma; Robert Warmerdam; Soesma Medema-Jankipersadsing.

For peer review only - http://bmjopen.bmj.com/site/about/guidelines.xhtml

Abstract

Objectives

Studies in clinical settings showed a potential relationship between Socio-Economic Status (SES) and lifestyle factors with COVID-19, but it is still unknown whether this holds in the general population. In this study we investigated the associations of SES with self-reported, tested, and diagnosed COVID-19 status in the general population.

Design, setting, participants and outcome measures

Participants were 49,474 men and women (46 ± 12 yrs) residing in the Northern Netherlands from the Lifelines cohort study. SES indicators and lifestyle factors (i.e., smoking status, physical activity, alcohol intake, diet quality, sleep time, and TV watching time) were assessed by questionnaire from the Lifelines Biobank. Self-reported, tested, and diagnosed COVID-19 status were obtained from the Lifelines COVID-19 questionnaire.

Results

There were 4,711 participants who self-reported having had a COVID-19 infection, 2,883 participants tested for COVID-19, and 123 positive cases diagnosed in this study population. After adjustment for age, sex, lifestyle factors, BMI, and ethnicity, we found that participants with low education or low income were less likely to self-report a COVID-19 infection (OR [95%CI]: low education 0.78 [0.71-0.86]; low income 0.86 [0.79-0.93]), and be tested for COVID-19 (OR [95%CI]: low education 0.58 [0.52-0.66]; low income 0.86 [0.78-0.95]) compared with high education or high income groups, respectively.

Conclusion

Our findings suggest that the low SES group was the most vulnerable population to self-reported and tested COVID-19 status in the general population.

For peer review only - http://bmjopen.bmj.com/site/about/guidelines.xhtml

Strengths and limitations of this study

- This study added evidence to the socio-economically patterned COVID-19 status in a general population instead of in clinical settings.
- This study innovatively included a broader range of COVID-19 status, including self-reported and tested COVID-19 status, to better understand COVID-19 related socio-economic factors.
- This study might fail to identify the ethnic differences because the study population was predominantly white (more than 98%).
- During the first wave of the COVID-19 pandemic in the Northern Netherlands, the number of cases identified were relatively low compared with the rest of the country, which might limit the power of the analysis about diagnosed COVID-19 status.

For peer review only

Introduction

The ongoing COVID-19 pandemic has already infected 62,363,527 people worldwide (as of December 1, 2020) [1]. Many studies have reported a relationship between lifestyle factors (e.g., smoking [2] and unhealthy diets [3]) or comorbidities associated with lifestyle factors (e.g., obesity [4] and diabetes mellitus [5]), and worse outcomes for COVID-19. Since lifestyle factors are often associated with Socio-Economic Status (SES) [6], and several studies have revealed that people with low SES are more susceptible to COVID-19 infection, hospitalization, and mortality [7-11], the interplay between SES, lifestyle and COVID-19 status becomes a valid query and it is likely that SES is a determinant of COVID-19.

Therefore, a rising concern among policy makers and public health practitioners is that the COVID-19 pandemic might exacerbate the persistent socio-economically patterned health inequalities over the life course [12, 13]. Still, an in-depth investigation of the lifestyle and socio-economic determinants of COVID-19 in the general population is needed because current COVID-19 studies mainly relied on individuals presenting with symptoms in a clinical setting and focused on post COVID-19 infection status (e.g., hospitalization and intensive care) [14, 15]. As such, we do not have enough knowledge on the validity of these early results for the general population.

This study aimed to investigate the extent to which SES and lifestyle factors were associated with a broader range of COVID-19 status, i.e. self-reported COVID-19, tested COVID-19, and diagnosed COVID-19 in the general population.

Methods

Study design and participants

To assess COVID-19 status in the general population we used data from the Lifelines COVID-19 Cohort. The Lifelines COVID-19 Cohort is a questionnaire-based study in a part of the Dutch Lifelines cohort [16, 17]. To study the relationship between COVID-19 and SES, we linked data from the Lifelines COVID-19 questionnaire with data from the general Lifelines cohort, which contains a

For peer review only - http://bmjopen.bmj.com/site/about/guidelines.xhtml

wide range of background data, such as demographics, lifestyles, and SES [18, 19]. For the present study, 49,474 participants from both the Lifelines cohort and the Lifelines COVID-19 Cohort, who had available and reliable data on demographics, SES, and lifestyle were included in the analysis (supplementary figure 1).

The Lifelines cohort study is a multidisciplinary prospective population-based cohort study based on a unique three-generation design. The participants were recruited from the three Northern provinces of the Netherlands between 2006 and 2013, and the first group of participants were recruited via local general practitioners. The participants could indicate whether their family members were interested to be recruited as well. In addition, individuals who were interested in the study could also participant through online self-registration. Participants with insufficient knowledge of the Dutch language, with severe psychiatric or physical illness, and those with limited life expectancy (<5 years) were excluded from the study. The Lifelines cohort study employs a broad range of investigative procedures in assessing the biomedical, socio-demographic, behavioral, physical, and psychological factors which contribute to health and disease. A detailed description of the Lifelines cohort study can be found elsewhere [18, 19]. Before study entry, a signed informed consent form was obtained from each participant. Adult participants (≥ 18 years) were asked to complete several questionnaires regarding various aspects, including demographics, socio-economic status, and lifestyle. The Lifelines study is conducted according to the principles of the Declaration of Helsinki and approved by the Medical Ethics Committee of the University Medical Center Groningen, The Netherlands (2007/152).

The Lifelines COVID-19 Cohort collected data about COVID-19-related symptoms, current health issue and societal impacts from participants recruited from the Lifelines cohort. It is developed based on a COVID-19 questionnaire to identify genetic and environmental risk factors for COVID-19 and address the medical, social and psychological aspects of the pandemic. A detailed description of the Lifelines COVID-19 Cohort and the COVID-19 questionnaire can be found elsewhere [16]. The COVID-19 questionnaire is sent out weekly since March 30, 2020 for 12 weeks. Of the 139,713 Lifelines participants invited, 74,268 (53.2%) completed at least one of the questionnaires in the 12-week program.

For peer review only - http://bmjopen.bmj.com/site/about/guidelines.xhtml

COVID-19 Status

There were three different COVID-19 status obtained in our study from the COVID-19 questionnaire: self-reported COVID-19, tested COVID-19, and diagnosed COVID-19. All COVID-19 status was coded as binary variables. Self-reported COVID-19 status was obtained by asking “if you must choose, do you think you have (or have had) a COVID-19 infection?” or “has a doctor told you that you probably have (had) a coronavirus/COVID-19 infection”; tested COVID-19 status was obtained from the question “have you been tested for coronavirus (COVID-19)?”; diagnosed COVID-19 status was defined by asking “do you have or have you had a coronavirus/covid-19 infection?” or “what was the results of your corona virus (COVID-19) test?” when they had a COVID-19 test.

Since some of the COVID-related questions were worded such that it is not possible to infer whether the answer referred to the period between the last questionnaire and the current questionnaire, or to the beginning of the pandemic and the current questionnaire (e.g., “have you been tested for coronavirus (COVID-19)?”), the 12 questionnaires were condensed into a single observation per individual, indicating for each question whether the individual had at any point answered ‘yes’ during the study period.

Socio-Economic Status

Socio-Economic Status was indicated by a) highest educational level achieved and b) monthly net household income level separately from the Lifelines cohort study at baseline. Highest educational level achieved was categorized as: (1) low - junior general secondary education or lower (International Standard Classification of Education (ISCED) level 0, 1 or 2); (2) middle - secondary vocational education and senior general secondary education (ISCED level 3 or 4); and (3) high - higher vocational education or university (ISCED level 5 or 6) [20]; household net income level was categorized as: (1) low: <2000 euro/month; (2) middle: 2000-3000 euro/month; and (3) high: >3000 euro/month.

Lifestyle factors

For peer review only

Six lifestyle factors (i.e., smoking status, alcohol consumption, diet quality, physical activity, TV watching time, and sleep time) were selected from the Lifelines cohort study at baseline. Smoking status was categorized into never, former, and current smoker. Alcohol intake and dietary consumption were derived from a validated 110-item semi-quantitative food frequency questionnaire (FFQ) that assessed food consumption over the past month [21]. Heavy drinking was defined as >40 g or >20 g average per day alcohol consumption for men and women, respectively [22]. Lifelines Diet Score (LLDS) was calculated to assess the overall diet quality based on the FFQ. This score ranks the relative intake of nine food groups with positive health effects (vegetables, fruit, whole grain products, legumes/nuts, fish, oils/soft margarines, unsweetened dairy, coffee, and tea) and three food groups with negative health effects (red/processed meat, butter/hard margarines, and sugar-sweetened beverages). The development of this score is described in detail elsewhere [23]. Non-occupational moderate-to-vigorous physical activity (MVPA) was calculated in minutes per week from the validated Short Questionnaire to ASsess Health-enhancing physical activity (SQUASH) data, which incorporated leisure time and commuting physical activities, including sports, at moderate (4.0-6.4 MET) to vigorous ($\geq$6.5 MET) intensity [24]. TV watching time and sleep time were self-reported. Body mass index (BMI) was calculated by dividing weight in kilograms by the square of height in meters. The BMI was additionally categorized into underweight (BMI < 18.5 kg/m^2), normal (18.5 $\leqslant$ BMI < 25 kg/m^2), overweight (25 $\leqslant$ BMI < 30 kg/m^2), and obese (BMI $\geqslant$ 30 kg/m^2) [25].

Statistical analysis

Nominal variables are presented as percentage (%). Continuous variables were shown as mean ± standard deviation (SD) or median (interquartile range [IQR]). P-values < 0.05 were considered statistically significant. To analyze the associations of SES (i.e., education and income) with self-reported and tested COVID-19 status we used logistic regression models to estimate odds ratios (OR). Furthermore, we fitted robust Poisson regression models to estimate relative risk (RR) for associations between SES and diagnosed COVID-19 since the number of individuals with diagnosed COVID-19 was relatively low in this population. For all regression models, SES indicators were first entered into the model plus age and sex (model 1), and then adjusted for other covariates (model 2: model 1 plus

For peer review only - http://bmjopen.bmj.com/site/about/guidelines.xhtml

For peer review only

six lifestyle factors; model 3: model 2 plus BMI; model 4: model 3 plus ethnicity). All statistical analyses were conducted using Stata, version 13.1 (StataCorp, Texas, USA) or RStudio, version 4.0 (RStudio PBC. Boston, USA). Sensitivity analyses were conducted by further adjusting for the total number of questionnaires filled by each participants (supplementary table 1), by comparing the characteristics of study population and excluded participants (supplementary table 2), and by treating lifestyle factors together as a mediator in the pathway between SES and COVID-19 status (supplementary table 3).

Patient and public involvement Patients and/or the public were not involved in the design, or conduct, or reporting, or dissemination plans of this research.

Results

Participant characteristics are shown in Table 1. The average age of the study population was 46 ± 12 years old and more women were recruited in this study (59.7%). More than 92% of the population characterized their ethnicity as white. The prevalence of overweight and obese were 40.0% and 14.5% respectively (Table 1). Out of the 49,474 participants that were recruited in both Lifelines cohort and Lifelines COVID-19 cohort, 4,711 participants self-reported a COVID-19 infection, while 2,833 participants reported having had an COVID-19 test, and 123 participants reported having had a positive outcome of the COVID-19 test (Table 1). Self-reported, tested, and diagnosed COVID-19 participants were more likely to be female, to have attained middle or high education, to have high income, and to have never smoked (Table 1).

The association between education and different COVID-19 status are presented in Table 2. Participants with low and middle education level were less likely to self-report COVID-19 (OR [95% CI]: low 0.78 [0.71-0.86]; middle 0.90 [0.84-0.97]), and be tested for COVID-19 (OR [95% CI]: low 0.58 [0.52-0.66]; middle 0.72 [0.66-0.79]) compared with highly educated participants after adjustment for age, sex, lifestyle factors, ethnicity, and BMI (Table 2). In addition, compared with high education participants, participants with middle education level had almost two-fold higher risk

For peer review only - http://bmjopen.bmj.com/site/about/guidelines.xhtml

to be infected with COVID-19 (OR [95%]: 1.73 [1.19-2.50]) (Table 2, model 1) once they had been tested for COVID-19. The association was slightly attenuated after adjustment for lifestyle factors (OR [95%]: 1.69 [1.17-2.44]) (Table 2, model 2) and lifestyle factors seemed to slightly mediate the association between self-reported COVID-19 status (proportion mediated: 9.9%) (supplementary table 3), however, further adjustment for BMI and ethnicity did not change the results.

The association between income and different COVID-19 status are presented in Table 3. Low or middle income groups were less likely to self-report COVID-19 (OR [95% CI]: low 0.86 [0.79-0.93]; middle 0.80 [0.74-0.87]), and to be tested for COVID-19 (OR [95% CI]: low 0.86 [0.78-0.95]; middle 0.86 [0.78-0.94]) compared with high income group after adjustment for all covariates (table 3, model 4). Moreover, lifestyle factors only slightly mediated the association between self-reported, and tested COVID-19 status (proportion mediated: 6.7% and 9.5% respectively) (supplementary table 3). Nevertheless, no risk differences were found between different income levels and diagnosed COVID-19 status, once tested.

Discussion

This paper has provided evidence and insight for socio-economic disparities in self-reported COVID-19, tested COVID-19, and diagnosed COVID-19 in a general population. We found that low and middle SES groups, especially participants with low education, were less likely to self-report COVID-19 and to be tested for COVID-19. Moreover, participants with middle education were more likely to be infected with COVID-19 compared with those who obtained high education.

Our study highlighted the importance of mitigating the socio-economically patterned effect of the COVID-19 pandemic by showing a clear relationship between SES and COVID-19 status, which enables and aids policy makers to adequately respond to the pandemic and develop public health measurements. It is worrisome that low SES group is likely to be neglected or undetected during the pandemic because they are less likely to self-report and be tested for COVID-19. Given the fact that they are also at high risk of non-communicable diseases [26], once infected with COVID-19

For peer review only - http://bmjopen.bmj.com/site/about/guidelines.xhtml

or other infectious disease in the future, individuals with low SES might suffer disproportionately from the disease development compared with the high SES group, which may further exacerbate health inequality [27]. Therefore, national or regional commitment of public health measures, guidelines, or interventions are needed to promote health equity before, during, and after COVID-19 pandemic by identifying, targeting, and engaging the low SES group.

One of the potential mechanisms of the inverse association between SES and COVID-19 status is poor health literacy. Health literacy refers to “a person’s knowledge, motivation, and competences to access, understand , appraise, and apply health information in order to make judgments and take decisions in everyday life” [28]. It is known that low SES is the most important determinant of health literacy [29]. During the COVID-19 pandemic, people with high SES may be more aware of the symptoms and health consequences of COVID-19, and can, therefore, better interpret and communicate and make a decision about their own health condition [30, 31]. In other words, low SES people might be less sensitive and aware of their COVID-19 related symptoms, or they might lack the ability to assess if they could have had COVID-19 infection. Since they might lack health literacy, low SES people might be less likely to seek medical help even when they have symptoms because of the lack of practicing health seeking behavior and critical health resources [12, 13]. On the other hand, potentially low SES individuals had more difficulties obtaining a test even when they had sufficient health literacy. Our results also suggested that education level is a better determinant for COVID-19 status than income [29] because the association gradient was larger for low education participants than for low income participants. Further research is needed to fully understand the observed link between socio-economic disparities and COVID-19.

Furthermore, there could be other explanations of the inverse association of SES and COVID-19 status. Despite that fact that the low SES group is at higher risk of non-communicable disease, they also suffer from a higher general disease burden both mentally and financially during the pandemic [26]. Therefore, they might be less likely to notice the relevant symptom due to the already existing disease burden. Additionally, the first wave of COVID-19 pandemic in the Netherlands covered the winter holiday, and people with high income and education were more likely to go for a

For peer review only - http://bmjopen.bmj.com/site/about/guidelines.xhtml

For peer review only

relatively expensive skiing holiday compared to the low SES group. The skiing area in Italy has always been a popular destination for Dutch people and Northern Italy was the first outbreak center during the first wave of this pandemic [32], so that people with high income or high education who came back from the skiing holiday in Italy were more likely to self-report, and be tested for COVID-19.

Interestingly, lifestyle factors only slightly mediated the pathway between SES and self-reported, and tested COVID-19 status, and did not seem to be a mediator in the pathway between SES and diagnosed COVID-19 status in our study, which is different from a previous study that suggested an unhealthy lifestyle was a profound risk factor for COVID-19 status [15]. Some other studies also found that nutritional status [33, 34] and smoking [2] might also be risk factors for COVID-19 disease severity, disease progression, and mortality. Because of the differences of study population and COVID-19 outcome selection, our results were not comparable with previous studies. Nevertheless, this study provided a fresh message that in the general population, SES is a better determinant of self-reported, tested, and diagnosed COVID-19 status. In addition, unlike other COVID-19 studies that found obesity and ethnicity were both risk factors of COVID-19 infection, hospitalization, and mortality [11, 15, 35-38], our results showed that obesity and ethnicity did not contribute substantially to the disparities in COVID-19 status, which indicates that obesity and ethnicity were not in the pathway between SES and self-reported, tested, and diagnosed COVID-19 status.

This study has several strengths. First, this general population-based study about COVID-19 may to some extent mitigate the selection bias compared with studies conducted based in clinical settings and can allow us to thoroughly understand the relation between SES and COVID-19 in the general population.. Second, we innovatively included a broader range of COVID-19 status, including self-reported and tested COVID-19 status, to better understand COVID-19 related health literacy and accessibility to health services. Still, this study failed to link the objective measures of COVID-19 infection status, which could cause some bias. Another limitation of the current study is the fact that the study population was predominantly white (more than 98%). Additionally, the Netherlands has a well-developed social security system. This may limit its generalizability to populations of other

For peer review only - http://bmjopen.bmj.com/site/about/guidelines.xhtml

ethnicity, and in a different social context. One caveat of our study is that the relative low number of cases in the region, which is the nature of the COVID-19 outbreak in the Northern Netherlands (supplementary table 4), might influence the statistical power of the study. However, we still find statistically significant results for SES and COVID-19 status. Another limitation is from the condensed observations about COVID-19 status since certain types of individuals might be more likely to fill in more questionnaires, and are therefore more likely to report a positive COVID-19 status in at least one of the questionnaires. If these individuals predominantly belong to a specific SES group, this would bias the results. However, by further adjusting for the total number of questionnaires filled by each participants, we did not observe any difference from the current results (supplementary table 1). Additionally, although we only observed minor effect of lifestyle factor on the association between SES and COVID-19 status, the true effect of lifestyle factors could be more pronounced since the low SES group tends to report their lifestyle according to the social desire. Lastly, approximately 17% of the participants were dropped because of missing SES data, while 16% participants were dropped because of incomplete lifestyle information, which might bias the results given that people with low SES and worse lifestyle practices could be less likely to participant in the COVID-19 questionnaire. Nevertheless, both the study population and excluded participants have similar characteristics (supplementary table 2). Consequently, it is less likely that there was a selection bias resulting from missing variables.

In conclusion, our findings of SES and self-reported, tested, and diagnosed COVID-19 status indicate that individuals with lower SES may be more vulnerable and neglected during COVID-19 pandemic, possibly because of disease burden, poor health literacy and access to healthcare, suggesting that health services and health promotion interventions should particularly focus on targeting individuals with lower SES to get them better prepared for future health emergencies.

Contributions YZ: analysis design and execution, drafted the manuscript. MD: literature review, study design, manuscript review and approval. HD: study design, supervision, manuscript review and

For peer review only - http://bmjopen.bmj.com/site/about/guidelines.xhtml

For peer review only

approval. RDF: study design, supervision, manuscript review and approval. LHD: conception, supervision, manuscript review and approval. JOM and Lifelines Corona Research initiative: conception, manuscript review and approval, stakeholder engagement.

Acknowledgement The authors wish to acknowledge the services of the Lifelines cohort study, the contributing research centers delivering data to Lifelines, and all the study participants.

Funding This project has received funding from the European Union's Horizon 2020 research and innovation programme under the Marie Skłodowska-Curie grant agreement No 754425 as well as funding from NWO-Fast Track No 440.20.002. The Lifelines Biobank initiative has been made possible by funds from FES (Fonds Economische Structuurversterking), SNN (Samenwerkingsverband Noord Nederland) and REP (Ruimtelijk Economisch Programma).

Competing interests The authors have declared that no competing interests exist.

Patient and public involvement Patients and/or the public were not involved in the design, or conduct, or reporting, or dissemination plans of this research.

Ethics approval The Lifelines study is conducted according to the principles of the Declaration of Helsinki and approved by the Medical Ethics Committee of the University Medical Center Groningen, The Netherlands (2007/152). Before study entry, a signed informed consent form was obtained from each participant.

Data availability statement Data may be obtained from a third party and are not publicly available.

Reference

1. WHO. *WHO Coronavirus Disease (COVID-19) Dashboard*. 2020; Available from: https://covid19.who.int/?gclid=EAIaIQobChMIzsuel8uQ7AIVgrh3Ch2FbgICEAAYASAAEgL5y_D_BwE.
2. Vardavas, C.I. and K. Nikitara, *COVID-19 and smoking: A systematic review of the evidence.* Tobacco Induced Diseases, 2020. **18**(March).
3. Butler, M.J. and R.M. Barrientos, *The impact of nutrition on COVID-19 susceptibility and long-term consequences.* Brain, Behavior, and Immunity, 2020. **87**: p. 53-54.

For peer review only

For peer review only - http://bmjopen.bmj.com/site/about/guidelines.xhtml

4. Tartof, S.Y., et al., *Obesity and Mortality Among Patients Diagnosed With COVID-19: Results From an Integrated Health Care Organization.* Ann Intern Med, 2020.
5. Cuschieri, S. and S. Grech, *COVID-19 and diabetes: The why, the what and the how.* Journal of Diabetes and its Complications, 2020. **34**(9): p. 107637.
6. Glorioso, V. and M. Pisati, *Socioeconomic inequality in health-related behaviors: a lifestyle approach.* Quality & Quantity, 2014. **48**(5): p. 2859-2879.
7. Batty, G.D., et al., *Psychosocial factors and hospitalisations for COVID-19: Prospective cohort study based on a community sample.* Brain Behav Immun, 2020.
8. McCarthy, C.P., et al., *Early clinical and sociodemographic experience with patients hospitalized with COVID-19 at a large American healthcare system.* EClinicalMedicine, 2020: p. 100504.
9. Khalatbari-Soltani, S., et al., *Importance of collecting data on socioeconomic determinants from the early stage of the COVID-19 outbreak onwards.* J Epidemiol Community Health, 2020. **74**(8): p. 620-623.
10. Baena-Diez, J.M., et al., *Impact of COVID-19 outbreak by income: hitting hardest the most deprived.* J Public Health (Oxf), 2020.
11. Lieberman-Cribbin, W., et al., *Disparities in COVID-19 Testing and Positivity in New York City.* American Journal of Preventive Medicine, 2020. **59**(3): p. 326-332.
12. Burström, B. and W. Tao, *Social determinants of health and inequalities in COVID-19.* European Journal of Public Health, 2020. **30**(4): p. 617-618.
13. Schulz, A.J., et al., *Moving Health Education and Behavior Upstream: Lessons From COVID-19 for Addressing Structural Drivers of Health Inequities.* Health Educ Behav, 2020. **47**(4): p. 519-524.
14. Wynants, L., et al., *Prediction models for diagnosis and prognosis of covid-19: systematic review and critical appraisal.* BMJ, 2020. **369**: p. m1328.
15. Hamer, M., et al., *Lifestyle risk factors, inflammatory mechanisms, and COVID-19 hospitalization: A community-based cohort study of 387,109 adults in UK.* Brain, Behavior, and Immunity, 2020. **87**: p. 184-187.
16. Mc Intyre, K., et al., *The Lifelines COVID-19 Cohort: a questionnaire-based study to investigate COVID-19 infection and its health and societal impacts in a Dutch population-based cohort.* medRxiv, 2020: p. 2020.06.19.20135426.
17. Varga, T.V., et al., *Loneliness, worries, anxiety, and precautionary behaviours in response to the COVID-19 pandemic: a longitudinal analysis of 200,000 Western and Northern Europeans.* The Lancet Regional Health - Europe, 2021.
18. Klijs, B., et al., *Representativeness of the LifeLines Cohort Study.* PLoS One, 2015. **10**(9): p. e0137203.
19. Scholtens, S., et al., *Cohort Profile: LifeLines, a three-generation cohort study and biobank.* International Journal of Epidemiology, 2014. **44**(4): p. 1172-1180.
20. *International standard classification of education*. 1997, United nations educational, scientific, and cultural organization.
21. Siebelink, E., A. Geelen, and J.H. de Vries, *Self-reported energy intake by FFQ compared with actual energy intake to maintain body weight in 516 adults.* Br J Nutr, 2011. **106**(2): p. 274-81.
22. Dawson, D.A., *Defining risk drinking.* Alcohol research & health : the journal of the National Institute on Alcohol Abuse and Alcoholism, 2011. **34**(2): p. 144-156.
23. Vinke, P.C., et al., *Development of the food-based Lifelines Diet Score (LLDS) and its application in 129,369 Lifelines participants.* Eur J Clin Nutr, 2018. **72**(8): p. 1111-1119.
24. Wendel-Vos, G.C.W., et al., *Reproducibility and relative validity of the short questionnaire to assess health-enhancing physical activity.* Journal of Clinical Epidemiology, 2003. **56**(12): p. 1163-1169.
25. WHO, *Obesity : preventing and managing the global epidemic : report of a WHO consultation*, in *WHO Technical Report Series*. 1999, World Health Organization: Geneva, Switzerland.

For peer review only - http://bmjopen.bmj.com/site/about/guidelines.xhtml

26. Collaborators, G.B.D.C.o.D., *Global, regional, and national age-sex-specific mortality for 282 causes of death in 195 countries and territories, 1980-2017: a systematic analysis for the Global Burden of Disease Study 2017.* Lancet (London, England), 2018. **392**(10159): p. 1736-1788.
27. Marmot, M. and J. Allen, *COVID-19: exposing and amplifying inequalities.* Journal of Epidemiology and Community Health, 2020. **74**(9): p. 681.
28. Sørensen, K., et al., *Health literacy and public health: A systematic review and integration of definitions and models.* BMC Public Health, 2012. **12**(1): p. 80.
29. Stormacq, C., S. Van den Broucke, and J. Wosinski, *Does health literacy mediate the relationship between socioeconomic status and health disparities? Integrative review.* Health Promotion International, 2019. **34**(5): p. e1-e17.
30. Zhong, B.L., et al., *Knowledge, attitudes, and practices towards COVID-19 among Chinese residents during the rapid rise period of the COVID-19 outbreak: a quick online cross-sectional survey.* Int J Biol Sci, 2020. **16**(10): p. 1745-1752.
31. Alobuia, W.M., et al., *Racial disparities in knowledge, attitudes and practices related to COVID-19 in the USA.* J Public Health (Oxf), 2020. **42**(3): p. 470-478.
32. Distante, C., P. Piscitelli, and A. Miani, *Covid-19 Outbreak Progression in Italian Regions: Approaching the Peak by the End of March in Northern Italy and First Week of April in Southern Italy.* International Journal of Environmental Research and Public Health, 2020. **17**(9).
33. Richardson, D.P. and J.A. Lovegrove, *Nutritional status of micronutrients as a possible and modifiable risk factor for COVID-19: a UK perspective.* British Journal of Nutrition, 2020: p. 1-7.
34. Zabetakis, I., et al., *COVID-19: The Inflammation Link and the Role of Nutrition in Potential Mitigation.* Nutrients, 2020. **12**(5).
35. Sattar, N., et al., *BMI and future risk for COVID-19 infection and death across sex, age and ethnicity: Preliminary findings from UK biobank.* Diabetes & Metabolic Syndrome: Clinical Research & Reviews, 2020. **14**(5): p. 1149-1151.
36. Patel, A.P., et al., *Race, socioeconomic deprivation, and hospitalization for COVID-19 in English participants of a national biobank.* Int J Equity Health, 2020. **19**(1): p. 114.
37. Raisi-Estabragh, Z., et al., *Greater risk of severe COVID-19 in Black, Asian and Minority Ethnic populations is not explained by cardiometabolic, socioeconomic or behavioural factors, or by 25(OH)-vitamin D status: study of 1326 cases from the UK Biobank.* J Public Health (Oxf), 2020. **42**(3): p. 451-460.
38. Adhikari, S., et al., *Assessment of Community-Level Disparities in Coronavirus Disease 2019 (COVID-19) Infections and Deaths in Large US Metropolitan Areas.* JAMA network open, 2020. **3**(7): p. e2016938-e2016938.

For peer review only - http://bmjopen.bmj.com/site/about/guidelines.xhtml

Table 1. Demographics, socio-economic status, and lifestyle of the study population

	COVID-19 Status			
	Self-reported (n=4711)	Tested (2883)	Diagnosed (123)	Total response (n=49,474)
Sex, male%	37.4	30.4	30.1	40.3
Age, mean ± sd	42 ± 11	44 ± 12	44 ± 9	46 ± 12
Ethnicity, white%	89.4	92.8	94.3	92.0
BMI, mean ± sd	25.9 ± 4.3	25.7 ± 4.3	25.7 ± 3.8	26.0 ± 4.2
Underweight,%	0.7	1.0	0.8	0.7
Normal,%	46.7	48.0	47.2	44.9
Overweight,%	37.2	36.6	40.7	40.0
Obese,%	15.4	14.5	11.4	14.5
Education,%				
Low	18.0	16.4	14.6	23.5
Middle	40.8	37.4	48.0	39.2
High	41.2	46.2	37.4	37.3
Income, %				
Low	30.1	28.3	23.6	28.1
Middle	29.5	30.7	33.3	33.7
High	40.4	41.0	43.1	38.2
Smoking, %				
Current	20.5	15.1	15.5	16.9
Former	33.0	36.1	40.7	36.3
Never	46.5	48.9	43.9	46.8
TV watching time ≥ 4h/day,%	15.2	15.1	18.7	16.7
Sleep time <7 or >9 h/day, %	16.3	14.4	13.8	14.9
MVPA<150 min/week,%	38.8	37.5	29.3	37.9
LLDS	24.3 ± 6.1	25.1 ± 6.0	25.4 ± 5.9	24.7 ± 6.0
Alcohol, g/day	4.9 (1.3-11.0)	3.9 (0.9-10.0)	3.5 (0.9-9.0)	4.9 (1.2-11.1)
Heavy drinker, %	3.1	2.8	2.4	2.6

*Abbreviations: *sd* standard deviation; *LLDS* lifelines diet score; *MVPA* non-occupational moderate-to-vigorous physical activity

Table 2. Association of education with different COVID-19 status

Education	COVID-19 Status					
	Self-reported		Tested		Diagnosed	
	OR [95%CI]	p	OR [95%CI]	p	RR [95%CI]	p
Model 1						
low	0.81 [0.75-0.89]	**<0.001**	0.58 [0.52-0.65]	**<0.001**	1.18 [0.67-2.07]	0.6
middle	0.93 [0.87-0.99]	**0.03**	0.73 [0.67-0.80]	**<0.001**	1.73 [1.19-2.50]	**0.004**
high			Ref			
Model 2						
low	0.79 [0.72-0.86]	**<0.001**	0.59 [0.53-0.66]	**<0.001**	1.11 [0.61-2.00]	0.7
middle	0.91 [0.85-0.97]	**0.006**	0.74 [0.68-0.81]	**<0.001**	1.69 [1.17-2.44]	**0.005**
high			Ref			
Model 3						
low	0.77 [0.71-0.85]	**<0.001**	0.59 [0.53-0.66]	**<0.001**	1.11 [0.61-2.00]	0.7

For peer review only

For peer review only - http://bmjopen.bmj.com/site/about/guidelines.xhtml

middle	0.90 [0.84-0.96]	**0.002**	0.74 [0.68-0.81]	**<0.001**	1.69 [1.16-2.45]	**0.006**
high			Ref			
Model 4						
low	0.78 [0.71-0.86]	**<0.001**	0.58 [0.52-0.66]	**<0.001**	1.10 [0.60-2.01]	0.8
middle	0.90 [0.84-0.97]	**0.004**	0.72 [0.66-0.79]	**<0.001**	1.77 [1.21-2.60]	**0.003**
high			Ref			

*Model 1: adjusted for education, age and sex. Model 2: adjusted for education, age, sex, and six lifestyle factors (smoking status, TV watching time ≥ 4h/day, sleep time <7 or >9 h/day, MVPA<150 min/week, LLDS, and heavy drinker). Model3: adjusted for education, age, sex, six lifestyle factors (smoking status, TV watching time ≥ 4h/day, sleep time <7 or >9 h/day, MVPA<150 min/week, LLDS, and heavy drinker), and BMI. Model 4: adjusted for education, age, sex, six lifestyle factors (smoking status, TV watching time ≥ 4h/day, sleep time <7 or >9 h/day, MVPA<150 min/week, LLDS, and heavy drinker), BMI, and ethnicity.

Table 3. Association of income with different COVID-19 status

Income	COVID-19 Status					
	Self-reported		Tested		Diagnosed	
	OR [95%CI]	p	OR [95%CI]	p	RR [95%CI]	p
Model 1						
low	0.89 [0.83-0.96]	**0.002**	0.85 [0.77-0.93]	**0.001**	0.84 [0.54-1.31]	0.4
middle	0.82 [0.76-0.88]	**<0.001**	0.84 [0.77-0.92]	**<0.001**	1.14 [0.77-1.70]	0.5
high			Ref			
Model 2						
low	0.88 [0.81-0.95]	**0.001**	0.87 [0.79-0.96]	**0.005**	0.80 [0.51-1.25]	0.3
middle	0.82 [0.76-0.88]	**<0.001**	0.86 [0.78-0.94]	**0.001**	1.10 [0.73-1.64]	0.6
high			Ref			
Model 3						
low	0.88 [0.81-0.95]	**0.001**	0.87 [0.79-0.96]	**0.006**	0.80 [0.51-1.25]	0.3
middle	0.81 [0.76-0.87]	**<0.001**	0.86 [0.78-0.94]	**0.001**	1.10 [0.73-1.64]	0.7
high			Ref			
Model 4						
low	0.86 [0.79-0.93]	**<0.001**	0.86 [0.78-0.95]	**0.002**	0.74 [0.47-1.18]	0.2
middle	0.80 [0.74-0.87]	**<0.001**	0.86 [0.78-0.94]	**0.002**	1.05 [0.70-1.57]	0.8
high			Ref			

*Model 1: adjusted for income, age and sex. Model 2: adjusted for income, age, sex, and six lifestyle factors (smoking status, TV watching time ≥ 4h/day, sleep time <7 or >9 h/day, MVPA<150 min/week, LLDS, and heavy drinker). Model3: adjusted for income, age, sex, six lifestyle factors (smoking status, TV watching time ≥ 4h/day, sleep time <7 or >9 h/day, MVPA<150 min/week, LLDS, and heavy drinker), and BMI. Model 4: adjusted for income, age, sex, six lifestyle factors (smoking status, TV watching time ≥ 4h/day, sleep time <7 or >9 h/day, MVPA<150 min/week, LLDS, and heavy drinker), BMI, and ethnicity.

For peer review only

For peer review only - http://bmjopen.bmj.com/site/about/guidelines.xhtml

Supplementary Figure 1. Study flow chart

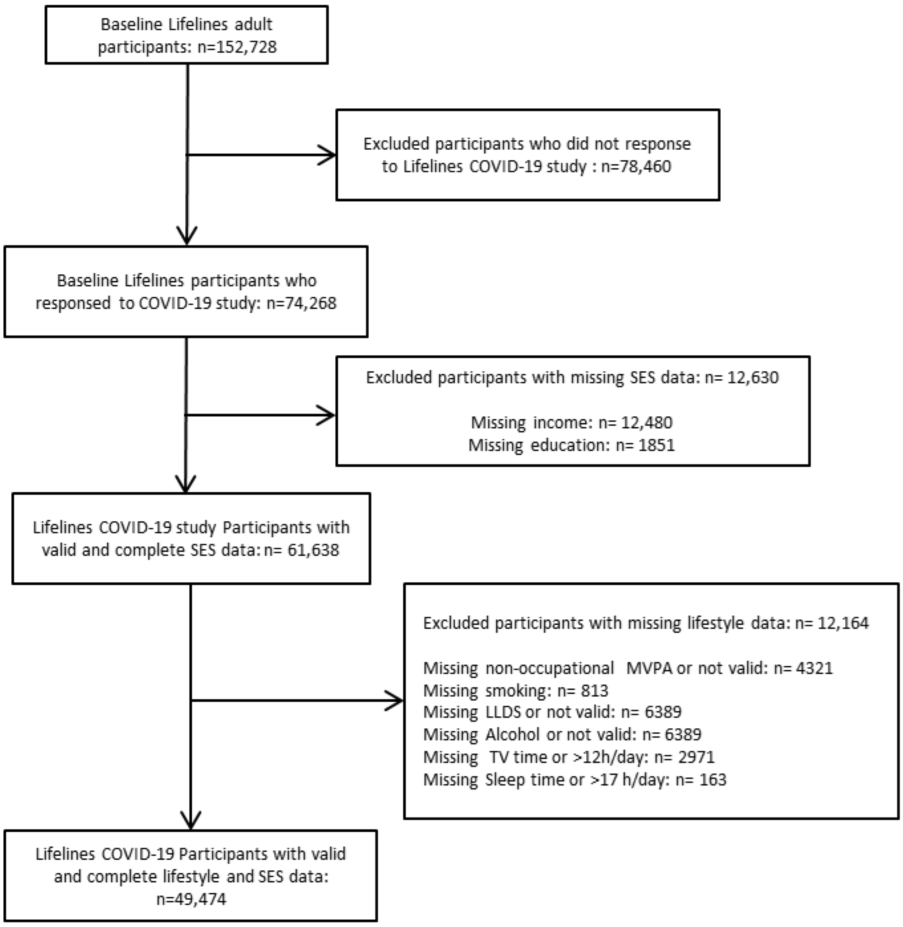

Supplementary Table 1. Association of education or income with COVID-19 status with further adjustment for number of questionnaires filled.

Education	COVID-19 Status					
	Self-reported		Tested		Diagnosed	
	OR [95%CI]	p	OR [95%CI]	p	RR [95%CI]	p
Model 1						
low	0.78 [0.71-0.86]	**<0.001**	0.58 [0.52-0.66]	**<0.001**	1.10 [0.60-2.01]	0.8
middle	0.90 [0.84-0.97]	**0.004**	0.72 [0.66-0.79]	**<0.001**	1.77 [1.21-2.60]	**0.003**
high			Ref			
Model 2						
low	0.79 [0.72-0.87]	**<0.001**	0.61 [0.54-0.68]	**<0.001**	1.08 [0.60-1.95]	0.8
middle	0.91 [0.84-0.97]	**0.006**	0.73 [0.67-0.80]	**<0.001**	1.72 [1.17-2.52]	**0.005**
high			Ref			
**Income**	**Self-reported**		**Tested**		**Diagnosed**	
	OR [95%CI]	p	OR [95%CI]	p	RR [95%CI]	p
Model 1						
low	0.86 [0.79-0.93]	**<0.001**	0.86 [0.78-0.95]	**0.002**	0.74 [0.47-1.18]	0.2
middle	0.80 [0.74-0.87]	**<0.001**	0.86 [0.78-0.94]	**0.002**	1.05 [0.70-1.57]	0.8
high			Ref			
Model 2						

view only

low	0.85 [0.78-0.92]	**<0.001**	0.84 [0.76-0.92]	**<0.001**	0.78 [0.49-1.24]	0.3
middle	0.80 [0.74-0.86]	**<0.001**	0.85 [0.77-0.93]	**0.001**	1.04 [0.69-1.55]	**0.9**
high			Ref			

*Model 1: adjusted for education or income, age, sex, six lifestyle factors (smoking status, TV watching time ≥ 4h/day, sleep time <7 or >9 h/day, MVPA<150 min/week, LLDS, and heavy drinker), BMI, and ethnicity. Model 2: adjusted for education or income, age, sex, six lifestyle factors (smoking status, TV watching time ≥ 4h/day, sleep time <7 or >9 h/day, MVPA<150 min/week, LLDS, and heavy drinker), BMI, ethnicity, and number of questionnaires filled.

Supplementary Table 2. Characteristics and COVID-19 status of study population and excluded population.

	Study population n= 49,474	Excluded population n= 24,794
Sex, male %	40.3	37.3
Age, mean (sd)	46 ± 12	45 ± 12
BMI, mean (sd)	26.0 ± 4.2	26.0 ± 4.3
Underweight,%	0.7	0.8
Normal,%	44.9	42.0
Overweight,%	40.0	36.9
Obese,%	14.5	14.0
Ethnicity, white %	92.0	81.3
Self-reported covid-19, n (%)	4711 (9.5)	2534 (10.2)
Tested covid-19, n (%)	2883 (5.8)	1284 (5.2)
Diagnosed covid-19, n (%)	123 (0.3)	50 (0.2)

Supplementary Table 3. Mediation effect of lifestyle factors in the association between SES and COVID-19 status.

**SES indicators**	COVID-19 Status		
	Self-reported	Tested	Diagnosed
Education			
Proportion mediated (%)*	9.9	ns	ns
Income			
Proportion mediated (%)	6.7	9.5	ns

*The proportion mediated was calculated when the mediation effect of lifestyle factors was significant. As shown in the table, lifestyle factors only slightly mediated the association of SES and self-reported, and tested COVID-19 status because the proportion mediated was really small (<10%). This was in accordance with our regression model2 that the coefficient was slighted attenuated after adjustment for lifestyle factors.

Supplementary Table 4. COVID-19 diagnosed cases for the Netherlands as whole and for the Northern Provinces (Lifelines population). Source: RIVM, downloaded from the CoronaWatchNL Github.

Region	Diagnosed (cummulative on 28th of June, 2020)
Netherlands	50,167
North NL	1529
Groningen	362
Frisland	634
Drenthe	533

For peer review only

STROBE Statement—checklist of items that should be included in reports of observational studies

	Item No	Recommendation	Page No
**Title and abstract**	1	(*a*) Indicate the study's design with a commonly used term in the title or the abstract	1
		(*b*) Provide in the abstract an informative and balanced summary of what was done and what was found	2
**Introduction**			
Background/rationale	2	Explain the scientific background and rationale for the investigation being reported	4
Objectives	3	State specific objectives, including any prespecified hypotheses	4
**Methods**			
Study design	4	Present key elements of study design early in the paper	5
Setting	5	Describe the setting, locations, and relevant dates, including periods of recruitment, exposure, follow-up, and data collection	5
Participants	6	(*a*) *Cohort study*—Give the eligibility criteria, and the sources and methods of selection of participants. Describe methods of follow-up<br>*Case-control study*—Give the eligibility criteria, and the sources and methods of case ascertainment and control selection. Give the rationale for the choice of cases and controls<br>*Cross-sectional study*—Give the eligibility criteria, and the sources and methods of selection of participants	5
		(*b*) *Cohort study*—For matched studies, give matching criteria and number of exposed and unexposed<br>*Case-control study*—For matched studies, give matching criteria and the number of controls per case	
Variables	7	Clearly define all outcomes, exposures, predictors, potential confounders, and effect modifiers. Give diagnostic criteria, if applicable	6-7
Data sources/ measurement	8*	For each variable of interest, give sources of data and details of methods of assessment (measurement). Describe comparability of assessment methods if there is more than one group	5-7
Bias	9	Describe any efforts to address potential sources of bias	11-12
Study size	10	Explain how the study size was arrived at	supplementary
Quantitative variables	11	Explain how quantitative variables were handled in the analyses. If applicable, describe which groupings were chosen and why	5-7
Statistical methods	12	(*a*) Describe all statistical methods, including those used to control for confounding	7-8
		(*b*) Describe any methods used to examine subgroups and interactions	7-8
		(*c*) Explain how missing data were addressed	5
		(*d*) *Cohort study*—If applicable, explain how loss to follow-up was addressed<br>*Case-control study*—If applicable, explain how matching of cases and controls was addressed<br>*Cross-sectional study*—If applicable, describe analytical methods taking account of sampling strategy	
		(*e*) Describe any sensitivity analyses	7-8

Continued on next page

For peer review only

For peer review only - http://bmjopen.bmj.com/site/about/guidelines.xhtml

**Results**			
Participants	13*	(a) Report numbers of individuals at each stage of study—eg numbers potentially eligible, examined for eligibility, confirmed eligible, included in the study, completing follow-up, and analysed	8
		(b) Give reasons for non-participation at each stage	
		(c) Consider use of a flow diagram	supplementary
Descriptive data	14*	(a) Give characteristics of study participants (eg demographic, clinical, social) and information on exposures and potential confounders	8
		(b) Indicate number of participants with missing data for each variable of interest	supplementary
		(c) *Cohort study*—Summarise follow-up time (eg, average and total amount)	
Outcome data	15*	*Cohort study*—Report numbers of outcome events or summary measures over time	
		Case-control study—Report numbers in each exposure category, or summary measures of exposure	
		Cross-sectional study—Report numbers of outcome events or summary measures	*8*
Main results	16	(*a*) Give unadjusted estimates and, if applicable, confounder-adjusted estimates and their precision (eg, 95% confidence interval). Make clear which confounders were adjusted for and why they were included	8-9
		(*b*) Report category boundaries when continuous variables were categorized	
		(*c*) If relevant, consider translating estimates of relative risk into absolute risk for a meaningful time period	
Other analyses	17	Report other analyses done—eg analyses of subgroups and interactions, and sensitivity analyses	11
**Discussion**			
Key results	18	Summarise key results with reference to study objectives	9
Limitations	19	Discuss limitations of the study, taking into account sources of potential bias or imprecision. Discuss both direction and magnitude of any potential bias	11
Interpretation	20	Give a cautious overall interpretation of results considering objectives, limitations, multiplicity of analyses, results from similar studies, and other relevant evidence	10
Generalisability	21	Discuss the generalisability (external validity) of the study results	9
**Other information**			
Funding	22	Give the source of funding and the role of the funders for the present study and, if applicable, for the original study on which the present article is based	12

*Give information separately for cases and controls in case-control studies and, if applicable, for exposed and unexposed groups in cohort and cross-sectional studies.

Note: An Explanation and Elaboration article discusses each checklist item and gives methodological background and published examples of transparent reporting. The STROBE checklist is best used in conjunction with this article (freely available on the Web sites of PLoS Medicine at http://www.plosmedicine.org/, Annals of Internal Medicine at http://www.annals.org/, and Epidemiology at http://www.epidem.com/). Information on the STROBE Initiative is available at www.strobe-statement.org.

For peer review only
